# Functional traits explain responses of Appalachian birds to vertical and horizontal forest structure

**DOI:** 10.1111/1365-2656.70326

**Published:** 2026-07-31

**Authors:** Trevor Roberts, Marta A. Jarzyna, Chenyang Wei, Colin P. Sweeney, Hikaru Keebler, Daniel Fink, Kaiguang Zhao, Benjamin Zuckerberg

**Affiliations:** ^1^ Department of Forest and Wildlife Ecology University of Wisconsin‐Madison Madison Wisconsin USA; ^2^ Department of Evolution, Ecology and Organismal Biology The Ohio State University Columbus Ohio USA; ^3^ Translational Data Analytics Institute The Ohio State University Columbus Ohio USA; ^4^ Lab of Ornithology Cornell University Ithaca New York USA; ^5^ School of Environment and Natural Resources The Ohio State University Columbus Ohio USA

**Keywords:** Appalachia, birds, eBird, forest structure, functional traits, hierarchical modelling, LiDAR

## Abstract

1. Species interact with habitat based on their functional traits, but it is unclear how specific traits interact with the multiple components of habitat structure.
2. To address this knowledge gap for forest‐dwelling bird communities, we leveraged forest structure information from NASA's Global Ecosystems Dynamics Investigation (GEDI) spaceborne light detection and ranging (LiDAR) mission alongside broad‐scale observations of bird occurrence from eBird. Using a custom implementation of the hierarchical modelling of species community (HMSC) approach, we estimated relationships between functional traits and the responses of bird populations to vertical and horizontal forest structure.
3. Although variable importance indicated that climate and elevation explained the most variation in bird occurrence, we discovered two strong signals of trait interactions with species' responses to forest structure: specialized foraging strategies explained birds' responses to vertical structure, while larger bodied birds with high flight efficiency showed weaker responses to both vertical and horizontal structure. Other functional traits such as diet and nest height were also associated with responses to vertical and horizontal structure, but with greater uncertainty.
4. Our results demonstrate the explanatory power of functional traits in describing birds' interactions with habitat and suggest that functional traits may play a role in structuring species' responses to forest structure.
5. The use of broad‐scale forest structure and bird occurrence data clarifies the impact of behaviour and morphology on habitat use, better quantifying the relationships between birds and their environment.

## Graphical abstract

The study of bird–forest relationships dates back decades, but rarely have the relationships of functional traits to 3D forest structure responses been considered for dozens of species at regional scales.

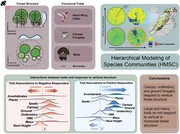

## 1 INTRODUCTION

The role of habitat structure in shaping species' niches has long fascinated ecologists (Dunlavy, 1935; Müller et al., 2010; Pickett & Siriwardena, 2011). Seminal research by MacArthur, for example, investigated relationships between habitat structure and the niches of forest birds (MacArthur, 1958; MacArthur & MacArthur, 1961). Habitat structure can refer to a wide variety of measures involving soils, disturbance and vegetation (Davies & Asner, 2014), but here we use structure to broadly refer to the physical arrangement, volume and identity of vegetation (Davies & Asner, 2014; LaRue et al., 2023). Previous work has demonstrated the importance of structure to the life histories of various taxa, including arthropods (Müller et al., 2014), non‐volant mammals (Palminteri et al., 2012) and especially birds (Davies & Asner, 2014; MacArthur, 1958). Many aspects of structure have well‐documented impacts on ecosystems and organisms. Prevalence of forest edge, for example, decreases the availability of microclimates in forest patches (Chen et al., 1993), while variation in the arrangement, volume and identity of vegetation tends to expand the niche space available to forest‐dwelling organisms (Dunlavy, 1935; Gámez & Harris, 2022).

**FIGURE 1:**
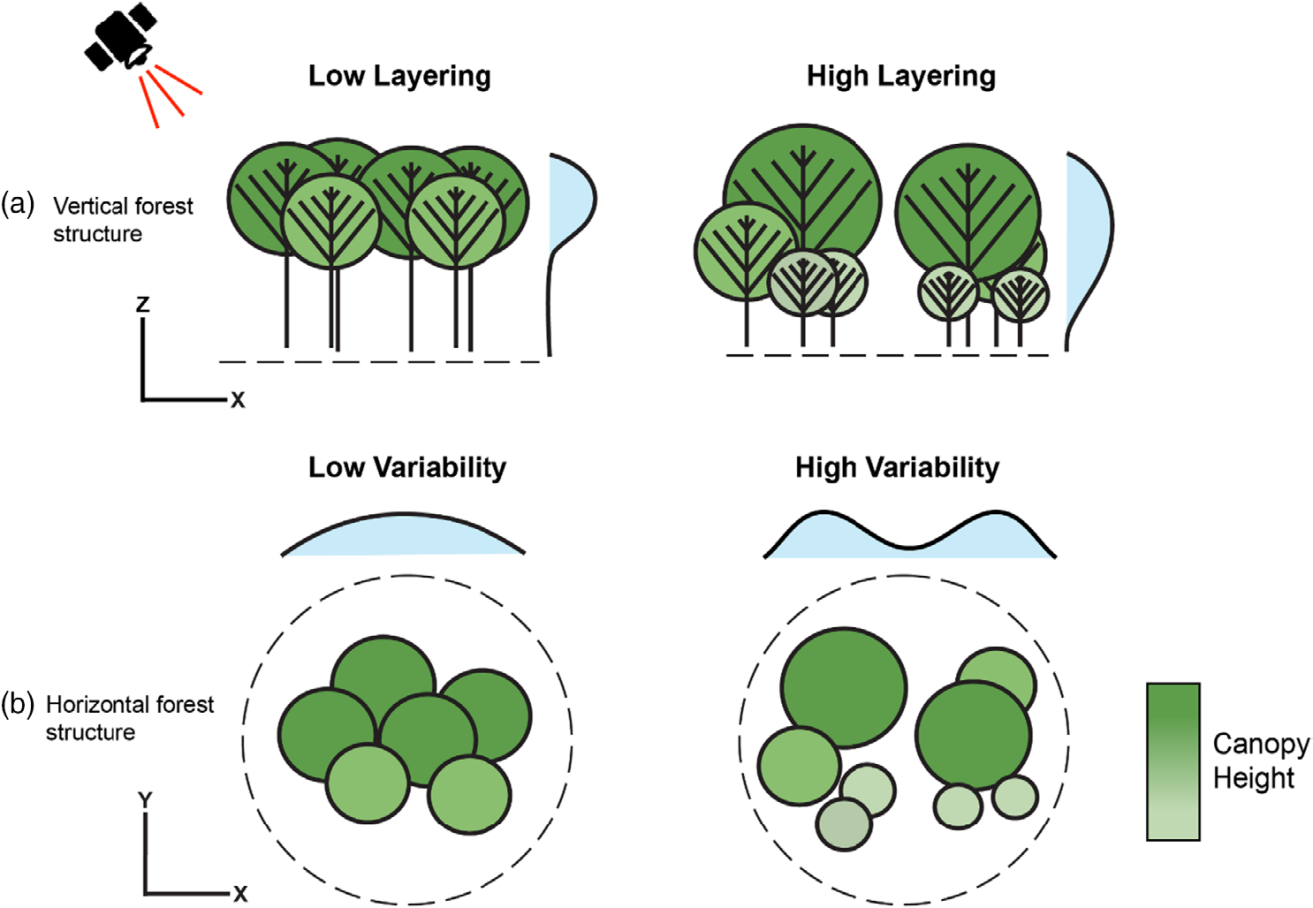
Separation of overall forest structure into its vertical and horizontal components at high and low values. Filled circles represent tree canopies. Darker shades of green indicate taller trees. (a) Cross‐sectional view of forest. Vertical forest structure captures variation in the X and Z planes, thus measuring the distribution of vegetation from ground to canopy. Low vertical structure means vegetation is concentrated in few canopy layers; high vertical structure means vegetation is distributed across vertical layers. (b) Top‐down view of forest. Horizontal forest structure captures variation in the X and Y planes, measuring vegetation distribution from tree‐to‐tree, within, and between forest patches. In this analysis, we define horizontal forest structure as variability in vegetation layering (i.e. vertical structure) across horizontal space. Therefore, low horizontal structure implies low variability in vegetation layering across horizontal space, and high horizontal structure implies high variability in vegetation layering across horizontal space. We used foliage height diversity (FHD) to capture vegetation layering and to quantify vertical and horizontal forest structure.

Although structure can be described for any habitat, forests are exemplary systems for studying habitat structure given the layering of vegetation from forest understorey to canopy (Carrasco et al., 2019; Davies & Asner, 2014). In forests, structure is readily separated into its horizontal (XY; e.g. forest extent and coverage) and vertical components (XZ; e.g. canopy height; Figure 1; Carrasco et al., 2019; Gámez & Harris, 2022). One notable measure of vertical forest structure is foliage height diversity (FHD), which captures the entire vertical distribution of vegetation with a single metric (MacArthur & MacArthur, 1961; see Section 2). Although the ecological effects of horizontal and vertical structure have been amply explored in isolation—such as fragmentation effects (Latimer & Zuckerberg, 2017) or the impact of vertical layering of vegetation on species richness (Culbert et al., 2013)—rarely are the two components considered concurrently. Recent advances in remote sensing technology and applications have facilitated measurement of vertical and horizontal structure across large spatial extents (e.g. regional, continental, global; Culbert et al., 2013; Potapov et al., 2021). For example, modern spaceborne light detection and ranging (LiDAR) missions such as NASA's Global Ecosystem Dynamics Investigation (GEDI) have begun to provide near‐global extent information on forest structure (Burns et al., 2024; Dubayah et al., 2020) and are now an invaluable data source for adding vertical information to studies involving forest structure across broad regions. By leveraging structure data from GEDI, species–forest structure (both horizontal and vertical) relationships can be assessed at regional, continental and even global scales.

Although species–forest structure relationships have been studied for a variety of taxa (Basham & Scheffers, 2020; Gámez & Harris, 2022), birds are a natural fit for forest structure studies due to their ability to exploit structure from ground to canopy and across multiple forest patches (Davies & Asner, 2014). Birds exhibit exceptional vagility and rely on resources (e.g. insects or seeds) and space (e.g. nest sites) across the entire vertical component of forest structure (Davies & Asner, 2014). Likewise, birds are capable of rapidly moving within and between forest patches (Claramunt et al., 2022; Sheard et al., 2020), enhancing their use of horizontal space. Furthermore, bird occurrence data are easily accessible due to the widespread adoption of participatory science platforms such as eBird (Sullivan et al., 2014).

Previous work has investigated relationships between forest structure and avian diversity (Culbert et al., 2013; Farwell et al., 2021; Silveira et al., 2023), but inferences are limited by a focus on taxonomic diversity rather than avian function. The use of avian functional traits, rather than taxonomic identities, may broaden understanding of the mechanisms underlying avian habitat relationships. Functional traits are essential for governing how birds associate with forest structure (Davies & Asner, 2014; McGill et al., 2006; Mouillot et al., 2021) as they describe *how* organisms interact with forest structure through measurable morphological or behavioural characteristics (McGill et al., 2006; Pigot et al., 2020). Indeed, many avian functional traits are expected to be associated with vertical or horizontal forest structure. Nesting preference, for example, likely interacts with the vertical layering of forests because birds locate their nests in tree canopies, tree cavities, on the ground, and along other parts of the vertical component of forests (Weisberg et al., 2014). Other traits like hand‐wing index, a proxy of flight efficiency (Sheard et al., 2020), are potentially related to horizontal structure because a species with high flight efficiency is likely able to access various patches of habitat. These expected associations between functional traits and the components of forest structure provide a foundation for predicting which traits are most strongly related to vertical or horizontal structure.

Our goal is to quantify how functional traits interact with species–forest structure relationships across both the vertical and horizontal components of forest structure. To detect signals of functional trait interactions in bird populations at the landscape scale, we integrate a GEDI‐derived forest structure data product (Wei et al., 2026), created by our research team, alongside data from eBird. Because functional traits reflect the behavioural strategies and morphological constraints through which species make use of their habitat, we hypothesize that some functional traits strongly interact with birds' relationships to vertical and horizontal forest structure. Specifically, we predict that canopy foraging and canopy nesting species are more sensitive to vertical structure because such species rely on canopy features (Table 1), whereas larger bodied birds with greater flight efficiency are more sensitive to horizontal structure due to their larger ranges and greater ease of movement (Schoener, 1968; Table 1). By integrating our GEDI forest structure product with broad‐scale bird occurrence and trait data for 106 species, we detail relationships between functional traits in bird populations and forest structure for the entire forest‐dwelling Appalachian avifauna.

**TABLE 1:** Traits used in analysis, their source, type, meaning, predicted relationship to structure based on our a priori reasoning, an example bird with high trait values and ecological rationale for our predictions based on each example bird. *None* structure prediction indicates that we generated no predictions regarding the relationship of a trait to vertical or horizontal structure.

Trait	Source	Type	Meaning	Structure pred.	Example	Rationale
Invertebrates	Wilman et al. (2014)	Diet	Percent of diet composed of invertebrates	Unknown	Olive‐sided Flycatcher (*C. cooperi*)	None
Plants	Wilman et al. (2014)	Diet	Percent of diet composed of plants	Unknown	Purple Finch (*H. purpureus*)	None
Seeds	Wilman et al. (2014)	Diet	Percent of diet composed of seeds	Unknown	Pine Siskin (*S. pinus*)	None
Canopy	Wilman et al. (2014)	Forage Stratum	Percent of foraging time spent in canopy layer	Vertical	Red‐eyed Vireo (*V. olivaceus*)	Concentrates foraging activity on canopy and subcanopy vegetation^a^
Ground	Wilman et al. (2014)	Forage Stratum	Percent of foraging time spent in ground layer	Vertical (+ Horizontal for ground foragers)	Blue Grosbeak (*P. caerulea*)	Prefers open forest with reduced canopy cover, edge habitats; relies on mixture of shrubby and open micro‐habitats near ground to forage^b^; ground foragers may show strong relationships to both vertical and horizontal structure
Understory	Wilman et al. (2014)	Forage Stratum	Percent of foraging time spent in understorey layer	Vertical	Hooded Warbler (*S. citrina*)	Sings and forages in dense understorey layer and canopy gaps^c^
HWI	Tobias et al. (2022)	Size	Hand Wing Index; measure of wing aspect ratio and flight efficiency	Horizontal	Ruby‐throated Hummingbird (*A. colubris*)	Extremely mobile and efficient flyer; associated with denser forest and open woodlands; frequently chases territorial intruders^d^
Mass	Tobias et al. (2022)	Size	Mass in grams	Horizontal	Wild Turkey (*M. gallopavo*)	Preference for mature forest interspersed with openings^e^
Nest Height	Sheard et al. (2024)	Nest	Average nest height above ground in metres	Vertical	Pine Warbler (*S. pinus*)	Commonly position nests high in forest canopy (10–15 m), although exhibit some nest height flexibility^f^

^a^ Cimprich et al. (2020).

^b^ Lowther and Ingold (2020).

^c^ Mumme et al. (2023).

^d^ Weidensaul et al. (2020).

^e^ McRoberts et al. (2020).

^f^ Rodewald et al. (2020).

## 2 MATERIALS AND METHODS

### 2.1 Study area

**FIGURE 2:**
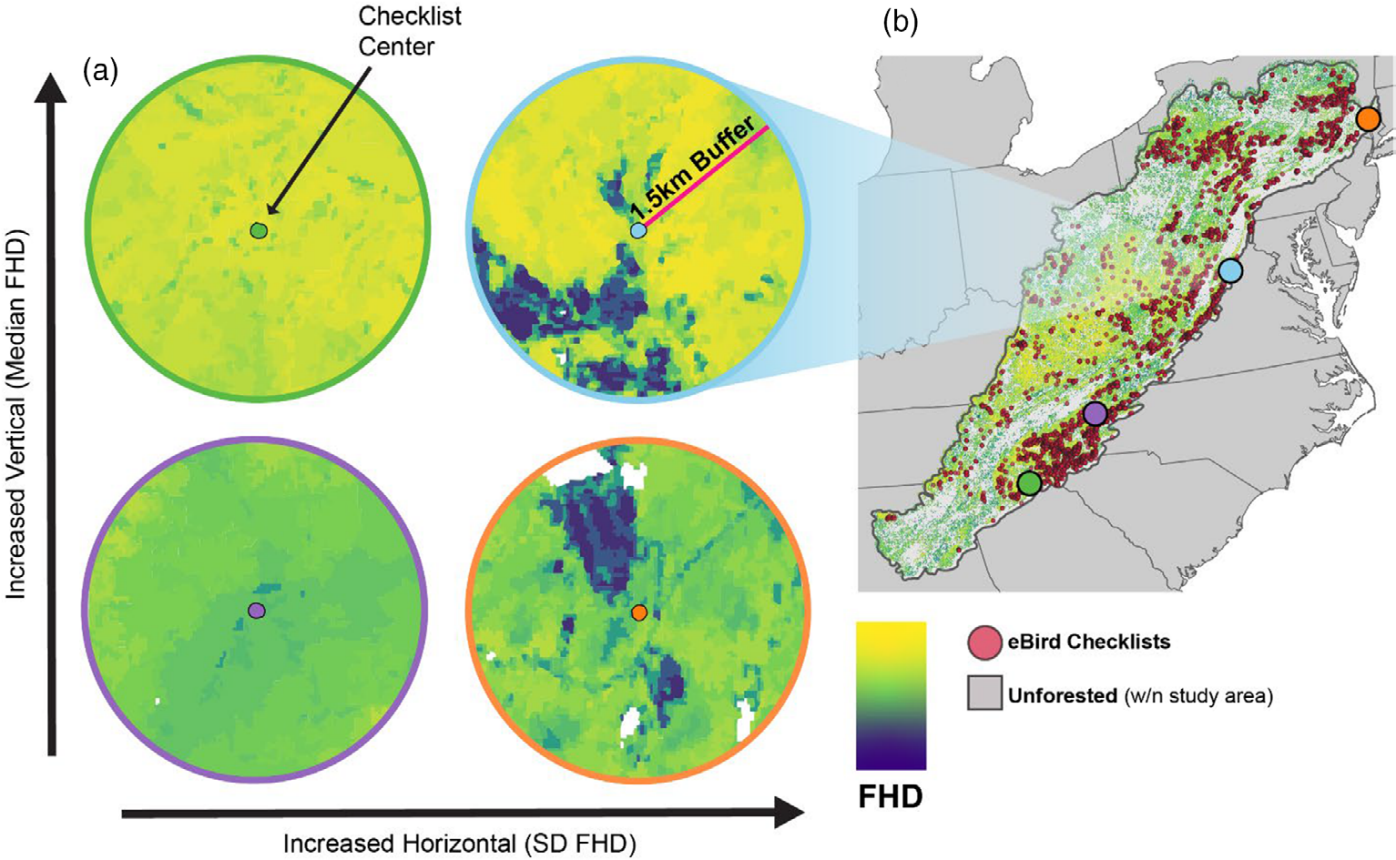
Foliage height diversity (FHD) values at four representative eBird checklists (i.e. observation location) in BCR28, Appalachia. (a) FHD is measured in 1.5 km buffers around checklist centres. Vertical structure is measured with median FHD; high values of median FHD indicate vertically structured vegetation (i.e. vertical layering; see vertical axis). Horizontal structure is quantified with standard deviation of FHD (SD FHD); high values of SD FHD indicate variation in FHD values across checklist buffers (see horizontal axis). (b) 6093 eBird checklists representing 106 bird species were filtered in BCR28 for the 2023 breeding season (1 May to 30 June). Coloured perimeters in (a) correspond to example points in (b). Points in (b) are not to scale.

Our analysis focused on the forest of the Appalachian Mountains (USA), which extends from northern Alabama to New England and are encompassed by Bird Conservation Region 28 (BCR28; Sauer et al., 2003; Figure 2). Preliminary analysis indicated that forests were tall (median canopy height = 24.6 m) and widespread (68.8% forest) in the region. We limited our analysis to one region to limit potential for spatial non‐stationarity in structure relationships across multiple regions. We analysed eBird, climate and elevation data from 2023, when available, to align data sources with the approximate collection window used to produce the GEDI product (2019–2022; Wei et al., 2026). Because all animal data were collected from a pre‐existing source, eBird, no ethical approvals were required to conduct this analysis. In 2023, the Appalachian Mountains (BCR28) had a gradient of average temperature ranging from 18.9°C in low‐lying, southern areas to 5°C at high elevations and in the northern part of the region. Total annual precipitation in 2023 was variable in the region, ranging from 624 to 2383 mm (Thornton et al., 2022). Appalachia sits at a relatively high elevation of 408 m on average, with substantial topographic relief (standard deviation = ±223 m). Elevation is generally higher and more varied in the eastern part of the region, gradually descending towards the western foothills (Dubayah et al., 2020).

### 2.2 Forest structure

We used data from GEDI LiDAR to quantify forest structure. GEDI is a spaceborne LiDAR system designed to measure forest structure at near global scale (Dubayah et al., 2020). Level 2 GEDI products (i.e. footprint level vegetation metrics) include various forest structure metrics, such as canopy height, canopy cover, plant area index (PAI) and multiple measures of vertical vegetation profile. However, these products consist of discontinuous 25 m diameter footprints (Dubayah et al., 2020) and are thus challenging to implement in wall‐to‐wall analyses of forest structure. Therefore, we created a new, fine‐grained (30 m) gridded product (details reported in Wei et al., 2026) and leveraged this product to extract forest structure information at eBird checklists (i.e. observation locations) across BCR28. Wei et al. (2026) created the forest structure product by relating GEDI data to 93 environmental covariates derived from various other remote sensing sources such as Landsat and Sentinel‐2. By tiling eastern North America into local (60 × 60 km) tiles prior to model fitting, random forest models achieved relatively accurate predictions of forest structure variables such as canopy height (median *R* ^2^ = 0.63) and FHD (median *R* ^2^ = 0.61).

We extracted foliage height diversity (FHD) from our GEDI product to measure vegetation structure with a single variable. FHD adapts the concept of Shannon entropy to foliage profiles (MacArthur & MacArthur, 1961; Shannon, 1948). In the case of GEDI, LiDAR waveforms are binned into 1 m increments from forest floor to upper canopy, then the function $-\sum_{i=1}^{S}\;p_{i}\times \log\left(p_{i}\right)$ is applied to the data where *S* equals the total number of 1‐m vertical height layers and *p*_*i*_ is the proportion of the total foliage area in the *i*‐th layer. Given this mathematical formulation, high values of FHD correspond to forests with vegetation distributed across vertical layers, while low FHD values indicate forests with vegetation concentrated in few vertical layers (Figure 1). Although our GEDI product included additional vertical structure metrics (e.g. canopy height, plant area index, etc.), we opted to use FHD given its explicit incorporation of all vegetation layers into a single, numerical estimate of the vertical vegetation profile as well as its importance in seminal research relating high FHD values to high levels of bird diversity (MacArthur & MacArthur, 1961). FHD alone does not describe all elements of forest structure, but as a highly researched, tractable metric of overall structure, it enabled us to model forest structure relationships across a large spatial extent for dozens of bird species. Furthermore, FHD estimation was more accurate in our fine‐grained (30 m) gridded product than many other metrics (Wei et al., 2026). We used FHD to quantify both vertical and horizontal forest structure. Vertical structure was quantified as the median FHD value in 1.5 km buffers around eBird checklists. We used FHD to quantify horizontal structure by summarizing variation in FHD across horizontal space (Figure 1). Specifically, we quantified horizontal structure as the standard deviation of FHD (SD FHD) in 1.5 km buffers (Figure 2). We restricted our analysis to relatively contiguous forests by filtering for eBird checklists with at least 80% forested pixels within 3 km buffers surrounding checklist centres; we defined forested pixels as those with >60% canopy cover and vegetation taller than 2 m. Canopy heights in filtered forests were tall (mean = 26.5 m; SD = 3.04 m). Thus, the filtering step limited comparisons to only tall, mature forests, excluding other habitat types which may have had different relationships between vegetation structure and species occurrence. This specific focus on mature forests allowed us to detect forest structure effects without conflating structure and habitat type. Within filtered forests, median FHD and SD FHD were not correlated to each other (*r* = 0.04) or other environmental predictors (e.g. temperature, precipitation, etc., see below).

### 2.3 Other environmental predictors

In addition to median FHD and SD FHD, we included elevation, landcover and climate information in our analysis to account for other important contextual factors of the local landscape and climate. We sourced elevation data from GEDI level 3 products (i.e. gridded metrics; Dubayah et al., 2020). Level 3 products were coarser than our forest structure data (1 km vs. 30 m) but still captured the broad‐scale effects of elevation. Small patches of missing data in the elevation product were interpolated using inverse distance weighting in the terra R package (Hijmans, 2022). Elevation values were assigned to eBird checklists by extracting the value from the 1 km pixel containing each checklist (Table S1).

Broadleaf deciduous trees compose most of the forests in BCR28, but needle‐leaved evergreen species can be locally dominant. To account for differences in forest composition, we used National Land Cover Database (NLCD) data to quantify the proportion of deciduous forest within 1.5‐km buffers surrounding eBird checklists (Homer et al., 2012). This strategy produced a metric ranging from zero to one, indexing forests with mostly deciduous trees (value close to one) or mostly evergreen trees (value close to zero). Deciduous forests made up ~76% of all forest lands in BCR28.

We used Daymet data from 2023 to produce climate predictors (Thornton et al., 2022). Although we modelled avian responses to forest structure over the breeding season, climatic conditions in other seasons are also relevant to overall habitat quality (Nott et al., 2002). Thus, we used annual values of temperature and precipitation to capture spatial variation in typical climate conditions across the study area. Total annual precipitation was extracted from the Daymet product based on the 1 km pixel in which each eBird checklist lay (Table S1). We extracted annual averages of maximum and minimum daily temperatures from Daymet also based on the position of each eBird checklist, then averaged these values to approximate mean annual temperature. All data extractions were completed in R 4.4 (R Core Team, 2024) using the terra and sf (Pebesma & Bivand, 2023) packages.

### 2.4 Bird occurrence

We used eBird for bird occurrence data across BCR28. eBird is a participatory science platform compiling user‐input data in semi‐structured checklists (Sullivan et al., 2014). eBird collects non‐detection records and ancillary information about search effort, facilitating the use of analytical methods designed for presence/absence data while accounting for important sources of variation in species detection rates (Johnston et al., 2021; Sullivan et al., 2014). We retained complete eBird checklists between 1 May and 30 June 2023, covering the breeding season for many species in BCR28. We chose to limit our analysis to the breeding season to focus exclusively on a single period during which relationships to structure should have remained stationary. Following established best practices to analyse eBird data (Johnston et al., 2021), we excluded checklists with >10 observers, >5 km of distance travelled and >5 h duration, and included only ‘travelling’ and ‘stationary’ type checklists. This initial filtering produced a dataset of 61,700 checklists.

Next, checklists were filtered to include only those in focal forests (see Section 2.2). We generated 3 km radial buffers centred on checklists and excluded those containing <80% forest pixels (as defined above). The 3 km buffers ensured that retained checklists were positioned in relatively intact forest tracts. For subsequent data extraction, we used a finer 1.5 km radius buffer to balance the relatively small breeding ranges of most forest‐dwelling birds (Sherry, 1979) with the nuances in eBird spatial accuracy. Although many bird species inhabit breeding territories smaller than 1.5 km radius, existing research indicates that species interact with environmental variation at scales four to nine times their median dispersal distance (Jackson & Fahrig, 2012). Therefore, buffers of 1.5 km radius should have captured relevant variation for any bird species that disperse at least 375 m (375 m × 4 = 1.5 km). Because even short dispersers tend to disperse at least 5 km (Chu & Claramunt, 2023), the scale of our analysis should have been sufficiently fine‐scale to model relationships between all analysed bird species and forest structure. Furthermore, previous work indicates that eBird spatial positioning data become unreliable below spatial resolutions of ~1.5 km (Strimas‐Mackey et al., 2020), so we determined that 1.5 km buffers were optimal to detect population‐level relationships with sufficient spatial accuracy (Strimas‐Mackey et al., 2020). Finally, occurrence observations were randomly sampled within a grid of 1.5 km width hexagons once per week, retaining a single checklist per week‐hexagon combination. Similar subsampling approaches are commonly used in eBird analyses to address site selection biases and to prevent strong clustering of observations in space and time (Fink et al., 2023; Johnston et al., 2019). Random realizations of this grid sampling approach did not appear to alter our results. In total, 6093 checklists met our strict thresholds for forest cover and independence (Figure 2).

The resulting data for BCR28 were distributed broadly across BCR28 and forests of varying structure (Figure 2) and hosted a large and diverse avifauna comprising 106 bird species spanning 27 families and nine orders. These bird species represented diverse traits and ecological strategies, from small, canopy foraging birds like Northern Parula (*Setophaga americana*) to large‐bodied, ground foragers like the Wild Turkey (*Meleagris gallopavo*). The substantial variation in climate, topography and forest structure in BCR28, alongside its diverse species pool, made it an excellent region for the study of trait interactions with forest structure responses.

### 2.5 Functional traits

We used three functional trait databases to extract species‐level information: AVONET (Tobias et al., 2022), which provides information on bird morphological traits; EltonTraits (Wilman et al., 2014) for foraging and dietary traits; and finally, a recent nest trait dataset for information on nest heights (Sheard et al., 2024). Combined, these three trait databases provided functional trait variables to test our predictions (Table 1).

Rather than include all possible functional traits in our analysis, we selected a subset of traits from the three trait databases based on our predictions of important trait–structure relationships (Table 1). Other traits not included in our selection also likely interact with forest structure relationships (e.g. territory size associations with horizontal structure relationships), but these traits were not included in the functional trait databases. Because the large species pool we analysed (106 species) necessitated compilation of large amounts of information using a systematic approach, we opted to limit our analysis to only pre‐existing trait data but acknowledge the opportunity to assess other important trait relationships. In particular, territory and home range size may have strongly interacted with horizontal structure relationships. However, traits included in our analysis (i.e. mass and HWI) are strongly correlated with territory size and should have therefore described much of the variation in species' use of horizontal space (Schoener, 1968; Thornton & Fletcher Jr., 2014; Tobias et al., 2022). We predicted that nest height and foraging behaviour explained birds' relationships to vertical structure (e.g. Pine Warbler [*Setophaga pinus*] and Red‐eyed Vireo [*Vireo olivaceus*]; Table 1), and that mass and HWI interacted with relationships to horizontal structure (e.g. Wild Turkey [*M. gallopavo*] and Ruby‐throated Hummingbird [*Archilochus colubris*]; Table 1). We also predicted that the diet of birds likely interacted with structure relationships, but we were unsure whether this interaction occurred predominately for vertical or horizontal structure. To quantify body size and wing dimensions, we used log‐transformed mass and hand wing index from AVONET; to quantify nest height, we used average nest heights above ground from the Sheard et al. database (Figure S1). Traits extracted from the EltonTraits database (foraging and dietary traits) were each composed of multiple axes. The diet trait category, for example, included 10 axes. We selected three axes each to represent the foraging behaviour and diet of the 106 bird species in our study area. To capture foraging behaviour, we chose canopy, understorey and ground foraging axes; to capture diet, we chose the invertebrates, plants and seeds axes. Although more axes were available to quantify foraging and diet, we chose to limit axes to those for which we had a priori predictions (Table 1). Furthermore, most birds in our study area primarily consumed invertebrates, vegetation, seeds or some combination of the three (84%); thus, our chosen axes captured most of the variation in diets across the regional avian species pool. Finally, we ensured all included traits were uncorrelated (*r* < 0.7; Dormann et al., 2013).

### 2.6 Phylogeny

Our analysis treated phylogeny as a potential source of non‐independence between observations. Therefore, we tested models with and without explicit incorporation of phylogenetic relatedness to determine whether inclusion of phylogenetic terms was necessary. To account for phylogenetic relationships, we extracted a pruned tree of the breeding bird species pool in BCR28 using the clootl package in R (Miller et al., 2025). The extracted tree integrated 67 published trees to quantify phylogenetic relationships (Table S2). We converted the pruned tree into a phylogenetic correlation matrix for use in downstream modelling.

### 2.7 Statistical modelling

We fit a two‐level hierarchical Bayesian model based on the Hierarchical Modelling of Species Communities (HMSC) framework (Ovaskainen et al., 2017) to estimate trait interactions with responses to forest structure (Equations (1), (2), (3), (4)). The first level of the model (Equations 1 and 2) regressed avian occurrence against environmental predictors, observation effort predictors and a term that captured remaining covariance in model residuals using two latent factors. Environmental predictors included temperature, precipitation, elevation, deciduous landcover composition and, most critically, vertical and horizontal forest structure variables (Table S1). Among the environmental predictors, we also included a species‐level intercept to account for baseline differences in each species' occurrence. We included effort variables such as ‘time spent birding’ to capture variability in the eBird observation process (Table S1). We chose not to model the observation process with an explicit detection submodel due to the difficulty of meeting occupancy modelling assumptions with eBird data and because eBird best practices indicate that the inclusion of effort covariates greatly improves prediction even without an explicit detection model (Cohen et al., 2023; Johnston et al., 2021). Although we chose not to explicitly model the observation process with a detection submodel, effort variables still captured substantial variation in detection rates as indicated by variable importance (Figure S2). All environmental and effort predictors were standardized to improve model convergence. Finally, the latent factors captured unexplained residual variation across species and checklists to account for species interactions and missing environmental predictors (Doser et al., 2023; Ovaskainen et al., 2017). We found that two latent factors captured significant variation, but that additional latent factors were insubstantial.

1. Occurrence submodel:

For sites $i=1,\ldots ,n_{\text{Checks}}$ and species $j=1,\ldots ,n_{\text{Species}}$:

(1) $$Z_{\mathrm{ij}}∼\text{Bernoulli}\left(\psi_{\mathrm{ij}}\right)$$

(2) $$\begin{array}{c} \text{logit}\left(\psi_{\mathrm{ij}}\right)=\underset{\text{environmental predictors}}{\underbrace{\sum_{b=1}^{n_{\text{Betas}}}B_{\mathrm{jb}}X_{\mathrm{ib}}}}+\underset{\text{effort predictors}}{\underbrace{\sum_{a=1}^{n_{\text{Alphas}}}A_{\mathrm{ja}}P_{\mathrm{ia}}}}+\underset{\text{latent factors}}{\underbrace{\sum_{k=1}^{n_{\text{Latent}}}U_{\mathrm{ik}}W_{\mathrm{jk}}}} \end{array}$$

Equations (1) and (2) to represent level one of the two‐level hierarchical Bayesian model. This level modelled avian occurrence (*Z*) at each checklist for each species with environmental predictors (*B*), effort predictors (*A*) and a latent factor term (*U × W*). Other terms include: Ψ, probability of occurrence; *X*, matrix of environmental predictors; *P*, matrix of effort predictors; *i*, checklist index; *j*, species index; *b*, environmental predictors index; *a*, effort predictors index; and *k*, latent factor index.

The second level of the model (Equations 3 and 4) estimated trait interactions by identifying traits that were systematically associated with the slope parameters describing species‐level associations with all the environmental predictors estimated in Equation (2). Trait data were standardized (i.e. *z*‐scored). Responses to environmental predictors (including species‐level intercepts) were modelled with a multivariate normal distribution to allow responses across predictors to inform each other.

2. Trait mediation submodel:

(3) $$\begin{array}{c} B_{j}=\left(\begin{array}{c} B_{j1}\\ \vdots \\ B_{j,n_{\text{Betas}}} \end{array}\right)∼\mathrm{MVN}\left(\mu_{B_{j}},\Sigma_{\beta }\right) \end{array}$$

with trait‐informed mean:

(4) $$\begin{array}{c} \mu_{B_{\mathrm{jb}}}=\;\Gamma_{0_{b}}+\sum_{t=1}^{n_{\text{Traits}}}\Gamma_{\mathrm{tb}}T_{\mathrm{jt}},\;b=1,\ldots ,n_{\text{Betas}} \end{array}$$

Equations (3) and (4) to represent level two of the two‐level hierarchical Bayesian model. This level modelled the responses to environmental predictors (*B*) for each species with corresponding trait predictors (*Γ*). Other terms include: *μ*_*β*_, average response across species to each environmental predictor; Σ_*β*_, covariance of species' responses to environmental predictors; Γ0, community trait intercept on environmental response; *T*, matrix of trait predictors; *j*, species index; *b*, environmental predictors index; *t*, traits index.

To ensure that underlying phylogenetic relationships did not affect our results, we fit an additional model to assess the impact of shared phylogeny on species' responses to environmental predictors (Figure S3). This model was identical to Equations ((1), (2), (3), (4)), except that a phylogenetic correlation matrix was included in the covariance of species' responses to environmental predictors (i.e. ∑_*β*_ in Equation 3). We tested the impact of underlying phylogenetic relationships on our results by comparing model coefficients and convergence between the models with and without the phylogenetic correlation matrix.

**FIGURE 3:**
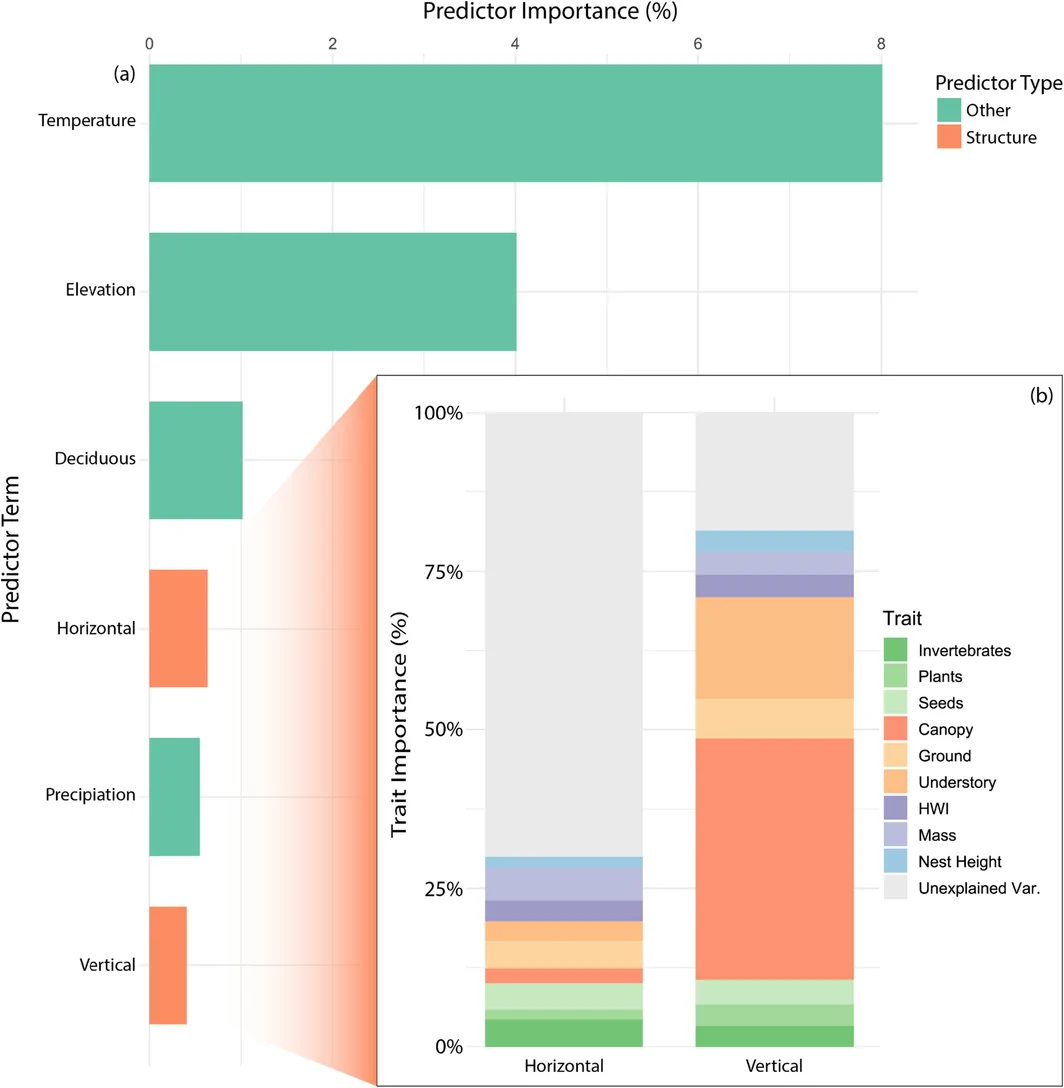
Variable importance for environmental (a) and trait (b) predictors in the two‐level Bayesian model for the entire forest avifauna of BCR28. Variable importance is expressed as percent variation explained by each predictor. (a) Structure predictors are highlighted in orange. Not all model predictors are included; thus, percentages do not sum to 100. (b) Stacked bars represent the importance of functional traits to the vertical and horizontal predictors expressed as percent variation explained. Traits of the same type are represented by similar colours.

Uninformative priors were applied to all estimated model parameters. Models were constructed and fit using the NIMBLE package in R (de Valpine et al., 2024). We ran three chains across 20,000 iterations with a 10,000 iteration burn‐in and a thinning factor of five to sample parameter posteriors. Model convergence was assessed with the potential scale reduction factor (Gelman & Rubin, 1992) in the coda R package (Plummer et al., 2006) and a convergence threshold of *R* < 1.05. If models failed to converge, we resampled posteriors across 200,000 iterations to ensure sufficient MCMC sampling occurred. We ran various diagnostics on the model output to ensure statistical validity of our results, including for spatial and phylogenetic autocorrelation (Pagel, 1999), correlations between parameter posteriors and general residual structure. To assess variable importance, we computed variance explained across both levels of the model (Figure 3).

### 2.8 Post hoc models

The two‐level hierarchical Bayesian model estimated associations between traits and structure responses across the entire forest avifauna of BCR28, but we were also interested in differences in trait effects among species with positive versus species with negative occurrence responses to forest structure. Because our primary hierarchical model was unable to estimate trait associations within subgroups of species, we also fit separate *post hoc* trait regression models of species' responses to vegetation structure for those species with positive occurrence responses and those species with negative occurrence responses. Species were classified as belonging to either the positive or negative response group if the 80% credible interval (CI) of a species' coefficient estimate *did not* overlap zero. We chose 80% CIs to increase admission to each group and reduce sensitivity of regressions to the exclusion of species (i.e. prioritized reducing Type II error).

All (positive and negative) response groups were subsets of the overall species pool and thus could have been sensitive to phylogenetic signals and multicollinearity not significant across the whole species pool. We tested for any phylogenetic signal in the response groups' forest structure coefficients using a simulation‐based test of Blomberg's *K* value (Blomberg et al., 2003) with the phytools R package (Revell, 2024). Positive and negative response groups with a significant phylogenetic signal were modelled with phylogenetic generalized linear models (Ho & Ane, 2014). We assessed trait multicollinearity in positive and negative response groups by using variance inflation factors (VIF). Trait predictors with VIF > 5 were considered multicollinear. We kept all traits in regressions but have indicated high VIF traits.

**FIGURE 4:**
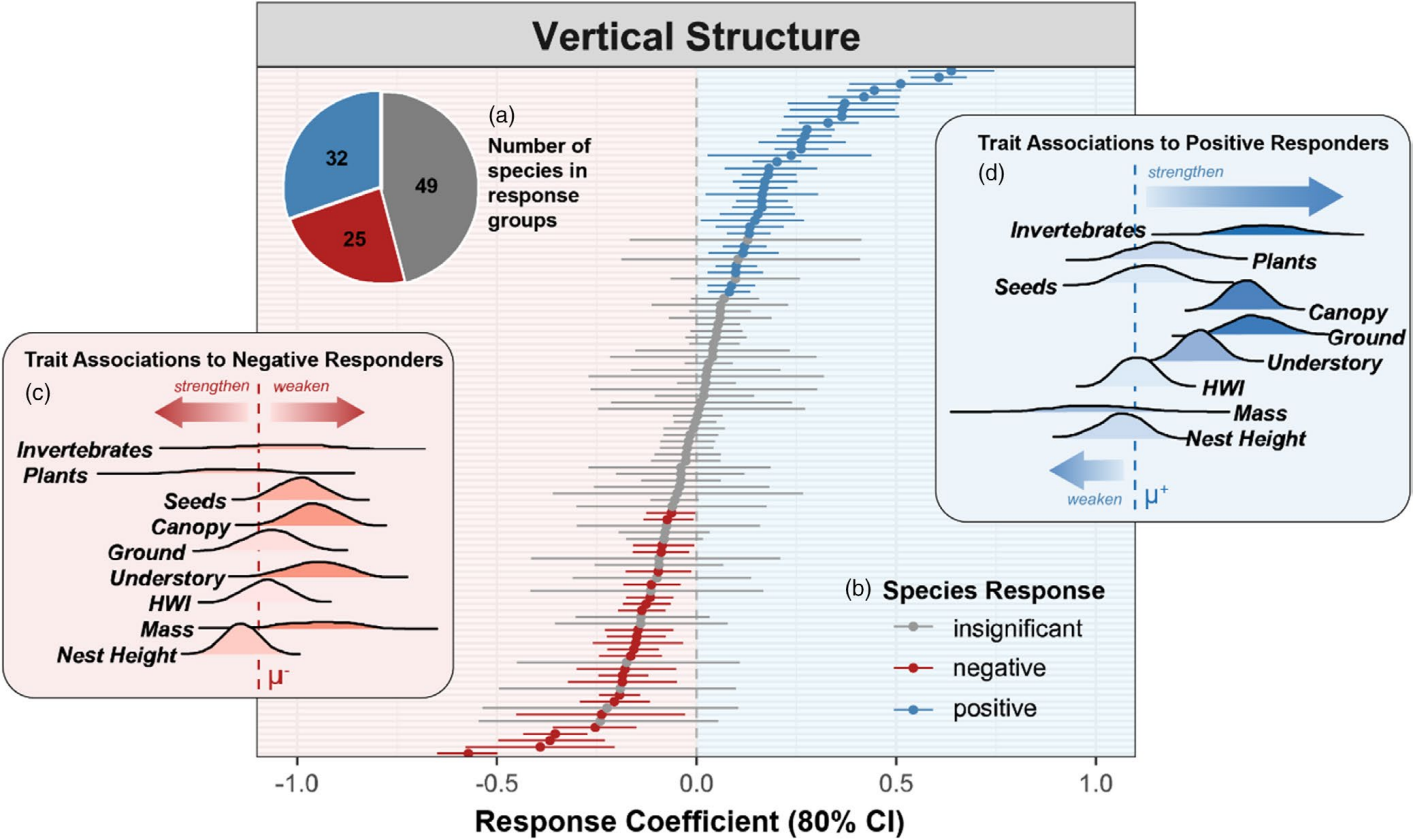
Species‐level responses to vertical forest structure (median FHD) for the BCR28 avian species pool from 1 May 2023 to 30 June 2023. (a) Pie chart represents number of species in positive response group (blue), negative response group (red) and insignificant response group (grey). (b) Caterpillar plot of species vertical structure coefficients and 80% CIs. (c, d) Ridge plots of trait effects for the negative (c) and positive (d) response groups. Density plots represent all 5999 resamples of model posteriors to estimate trait associations within response groups. Red and blue dashed lines represent the average response to vertical structure for negative and positive responders, respectively. Darker shading indicates less overlap of trait associations with zero. Arrows indicate ‘strengthening’ versus ‘weakening’ trait associations.

**FIGURE 5:**
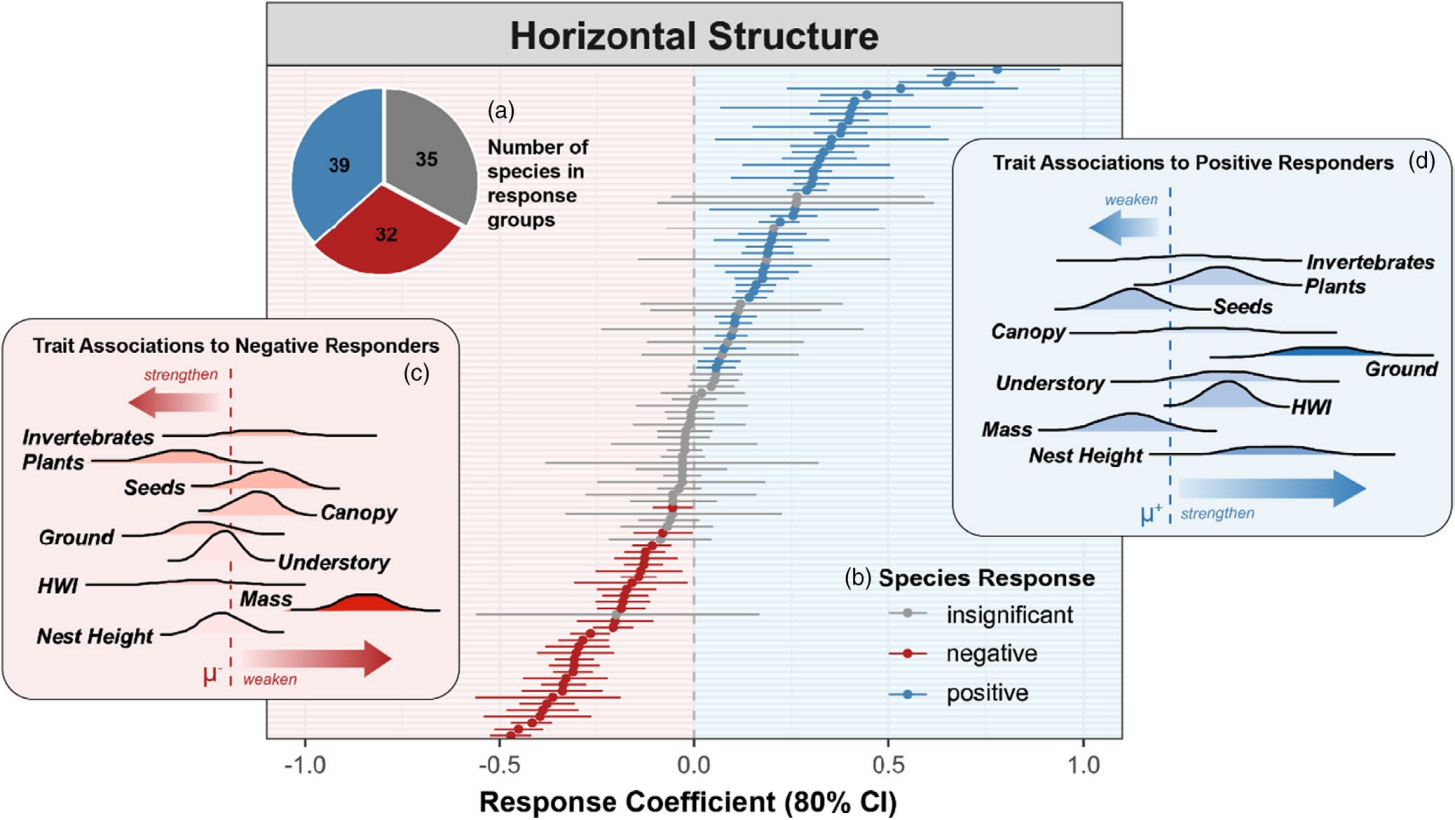
Species‐level responses to horizontal forest structure (standard deviation FHD) for the BCR28 avian species pool from 1 May 2023 to 30 June 2023. (a) Pie chart represents number of species in positive response group (blue), negative response group (red) and insignificant response group (grey). (b) Caterpillar plot of species horizontal structure coefficients and 80% CIs. (c, d) Ridge plots of trait effects for the negative (c) and positive (d) response groups. Density plots represent all 5999 resamples of model posteriors to estimate trait associations for response groups. Red and blue dashed lines represent the average response to horizontal structure for negative and positive responders, respectively. Darker shading indicates less overlap of associations with zero. Arrows indicate ‘strengthening’ versus ‘weakening’ trait associations.

To propagate uncertainty from the primary hierarchical model to the post hoc response group models, we resampled the posteriors of species' responses 5999 times then fit the trait regression model to each iteration. This resampling approach produced distributions of trait effects, which we visually inspected for dissimilarity from zero (Figures 4 and 5). We considered trait effects significant at two different levels: when 95% CIs did not overlap zero and a lower threshold for significance when 80% CIs did not overlap zero. Finally, we assessed trait interaction effects across positive and negative response groups, terming effects associated with amplified structure relationships (i.e. more negative or more positive) *strengthening* effects and those associated with dampened structure relationships (i.e. less negative or less positive) *weakening* effects (Table 2).

**TABLE 2:** Trait associations with response to each structure dimension in the negative and positive response groups.

Trait	Vertical structure (median FHD)		Horizontal structure (SD FHD)	
	Negative responders	Positive responders	Negative responders	Positive responders
Inverts.	NS	S	NS	NS
Plants	NS	NS	S	S
Seeds	W	NS	W	W
Canopy	W	S	W	NS
Ground	NS	S	NS	S
Understorey	W	S	NS	NS
HWI	NS	NS	NS	S
Mass	W	NS	W	W
Nest height	NS	NS	NS	S

*Note*: ‘S’ indicates a strengthening effect, either a more positive response (for positive responders) or a more negative response (for negative responders). ‘W’ indicates a weakening effect either a less positive response (for positive responders) or a less negative response (for negative responders). ‘NS’ indicates a non‐significant effect. Light green shading indicates a significant weakening effect at the 80% CI level, while dark green shading indicates the same for the 95% CI level. Light orange shading indicates a significant strengthening effect at the 80% CI level, and dark orange shading indicates the same for the 95% CI level.

### 2.9 Model diagnostics

Diagnostics of the two‐level Bayesian hierarchical model indicated that phylogenetic and spatial autocorrelation had minimal impact on our results, and thus, these terms were omitted from the final model. Furthermore, the latent factor terms captured residual variation without diminishing identification of environmental effects on occurrence (see ‘Model Diagnostics’ in supplement for more information; Figures S4 and S5).

## 3 RESULTS

### 3.1 Variable importance—Environment

We extracted variable importance from the first level (i.e. occurrence submodel) of the two‐level Bayesian model to assess the importance of predictors on species occurrence. Species‐level differences between baseline occurrence probabilities explained most of the variation in bird occurrence, as indicated by the importance of the model intercepts (68% of variation explained; Figure S2). The latent factors term explained an additional 7.85% of total explained variation. The remaining 22.5% of variation in occurrence was split between effort and environmental predictors (effort = 7.93%; environmental = 14.6%; Figure S2). Combined, SD FHD (i.e. horizontal predictor) and median FHD (i.e. vertical predictor) explained 1.0% of variation in occurrence, behind coarser scale environmental predictors like temperature (8.01%) and elevation (4.01%; Figure 3). SD FHD explained slightly more variation than median FHD (0.6% vs. 0.4%; Figure 3).

### 3.2 Variable importance—Traits

Trait importance extracted from the second level (i.e. trait mediation submodel) of the two‐level Bayesian model revealed which traits most explained variation in species' responses to environmental predictors. Traits explained large proportions of the variation in species' responses (i.e. variation in *B* parameters; Equations 3 and 4) to median FHD and SD FHD (Figure 3). 81% of variation in species' responses to median FHD was explained by traits, while 30% of variation in responses to SD FHD was explained by traits (Figure 3). For median FHD, forage stratum was the most important trait category of those tested (60% of variation explained), followed by diet (10.6%), then size and shape (7.2%) and nest height (3.3%). SD FHD showed less variation in trait importance, in which diet (10%), forage stratum (9.7%) and size and shape (8.4%) explained similar levels of variation, while nest height (1.7%) had minimal impact.

### 3.3 Trait interactions with species' responses to vertical structure

The average species' response to median FHD was not significantly different from zero (avg. response = 0.02 ± 0.04 95% CI). Slightly more species responded positively than negatively (32 vs. 25 species) to median FHD, while nearly half of species (49 species) had non‐significant responses (i.e. overlapped zero; Figure 4; Table S3; Figure S6). We used the *post hoc* trait regression models to assess which traits most interacted with species' occurrence responses in both the positive and negative response groups (Figure 4; Table S4). In the negative response group, occurrence of larger bodied birds that foraged for invertebrates and seeds in upper canopy layers was associated with weaker responses (less negative responses) to median FHD, while plant consumers, and, to a lesser extent, canopy nesters demonstrated stronger responses (more negative). Occurrence of ground foragers and high HWI birds had no discernible relationship to median FHD in the negative response group (Figure 4). In the positive response group, occurrence of invertebrate consumers and forage stratum specialists—canopy, ground or understory—was associated with stronger responses to median FHD (more positive). Occurrence of larger bodied birds and canopy nesters, on the other hand, was related to weaker (less positive) responses. Occurrence of plant and seed consumers and large‐winged birds showed no response to median FHD in the positive group (Figure 4; Table S4).

### 3.4 Trait interactions with species' responses to horizontal structure

The overall species pool response to SD FHD was slightly positive (avg. response = 0.04 ± 0.03 95% CI), indicating that occurrence increased in horizontally structured forests. However, species were split between positive (39), negative (32) and non‐significant (35) responses (Table S3; Figure S6). Like with vertical structure responses, the *post hoc* trait regression models revealed which functional traits were most associated with positive and negative occurrence responses to horizontal forest structure (Figure 5; Table S4). In the negative response group, responses in occurrence to SD FHD weakened (less negative) with increasing body mass, invertebrate and seed consumption, and canopy foraging. Occurrence of birds with plant diets, ground foraging and high hand wing index showed stronger (more negative) relationships to SD FHD. Meanwhile, occurrence of high nesting birds and understorey foragers in the negative response group showed negligible associations with SD FHD (Figure 5). In the positive response group, occurrence of ground foragers and high nesters strengthened (more positive) alongside SD FHD. Furthermore, occurrence of birds with high hand wing index, plant diets and canopy foraging strategies was associated with stronger responses (more positive), while occurrence of seed‐eating and large mass birds showed weaker responses (less positive) to SD FHD (Figure 5; Table S4).

### 3.5 Trait interactions across vertical and horizontal structure responses

We compared trait interactions across vertical and horizontal structure for both positive and negative response groups by compiling interactions fit from the *post hoc* trait regression models (Table 2). Trait effects largely differed between median FHD and SD FHD, but birds with large body mass consistently had weaker (either less positive or less negative) relationships to both components of forest structure (Table 2). The seed diet trait also demonstrated a weakening effect, meaning that seed eaters tended to have weaker relationships to median FHD and SD FHD. No other trait had consistent effects across responses to median FHD and SD FHD (Table 2).

## 4 DISCUSSION

The study of bird–forest relationships dates back decades, but rarely have the relationships of functional traits to forest structure responses been considered for dozens of species at regional scales (but see Culbert et al., 2013; Lesak et al., 2011; Xu et al., 2024). Given that bird traits impact fitness (Violle et al., 2007), the overall connection between traits and habitat use has important implications for characterizing how bird species make use of forests. We found that functional traits like foraging behaviour and body mass explain how birds interact with horizontal and vertical forest structure. In particular, where birds forage within forests largely explains their relationship to vertical structure while body size and flight efficiency are important determinants of responses to both vertical and horizontal structure.

### 4.1 Marginal importance of structure

Vertical (median FHD) and horizontal (SD FHD) forest structure metrics explained less variation in avian occurrence than predictors like temperature and elevation. Because we were interested in structural variation across relatively mature forests, we defined forests with strict height and vegetation cover filters. This filtering step limited inclusion of other habitat types in our analysis, as intended, but also limited variation of the forest structure predictors. Our forest structure predictors, in turn, were unable to explain as much variation in the region‐wide patterns of avian occurrence as more variable predictors like temperature. Still, forest structure predictors exhibited strong effects on the occurrence probabilities of some species, increasing and decreasing the probability of occurrence by up to 50%. Therefore, our results suggest that vertical and horizontal forest structure are still relevant components of habitat for the Appalachian avifauna despite lagging behind temperature and other predictors in variable importance. To further explore this point, we demonstrate how structure variables affect occurrence predictions at fine and coarse scales in the supplement (see Figures S7 and S8).

### 4.2 Vertical structure relationships

Most traits demonstrated similar variable importance for vertical and horizontal structure, except for traits describing foraging behaviour. For these traits—percent canopy, understorey or ground foraging—vertical structure associations were more important than horizontal associations. We predicted this disparity given the inherent impact foraging behaviour has on a bird's use of vertical space. Seminal studies in ecology and ornithology describe how birds partition niches along the vertical axis of forests by separating their diets and foraging strategies (Dunlavy, 1935; MacArthur, 1958). Therefore, the greater importance of foraging traits for describing variation in birds' responses to vertical structure is likely a signal of foraging strategy stratification from the forest floor to the upper canopy. Specifically, birds that were more strongly associated with vertically structured forests (i.e. high median FHD, many canopy layers) tended to specialize in canopy, understorey or ground foraging. These results suggest that foraging specialists, such as the Northern Parula (*S. americana*), rely on well‐developed vertical layering of forest canopy to procure sufficient resources, which is generally supported by the well‐documented sensitivity of forest specialists to degradation of intact, mature habitat (Callaghan et al., 2025). Although no other trait associations with structure responses were as strong as foraging, other traits like body mass (see below) and an invertebrate diet were still important. Invertebrate diet, in particular, exerted a strong strengthening effect on birds with positive relationships to vertical structure, implying that invertebrate consumers are more likely to occur in mature, vertically layered forests. Some existing evidence indicates that insect abundance increases in forests with greater canopy structural diversity (i.e. high median FHD) and closed canopy (Davies & Asner, 2014; Verdonck et al., 2025), and birds with an invertebrate diet preference, therefore, may be more likely to occupy vertically structured forests to take advantage of increased prey resources. Olive‐sided Flycatcher (*Contopus cooperi*), for example, sally for aerial insects from the uppermost branches of trees and, thus, benefit from vertically structured forests (Altman & Sallabanks, 2020). However, the responses of invertebrates to vertical structure are not uniform (Barbosa et al., 2005); some species‐level variation in the structure relationships of insectivorous birds is also expected.

### 4.3 Horizontal structure relationships

Traits were less important overall in describing the response of birds to horizontal structure. Furthermore, no single type of trait was substantially more important than any other. However, our prediction that mass and hand wing index would be especially important to horizontal structure was supported based on variable importance and the strong associations that mass had with responses to horizontal structure. Birds with high hand wing index were associated with stronger (more positive) relationships with horizontal structure. Because high hand wing index is indicative of flight efficiency and dispersal ability (Sheard et al., 2020; Tobias et al., 2022), birds with high hand wind index tend to inhabit larger ranges and, presumably, make use of a more spatially diffuse collection of resources (Schoener, 1968). Some Ruby‐throated Hummingbirds (*A. colubris*), a high HWI species, for example, exhibit a traplining foraging strategy in which they visit dispersed collections of flowers across landscapes (Feinsinger & Budd Chaplin, 1975). Bird species with large ranges or more diffuse foraging strategies may benefit from using multiple adjacent habitats to procure resources (i.e. complementary habitats; Mandelik et al., 2012) which may be reflected in positive relationships between occurrence and high horizontal structure (i.e. high SD FHD). Building upon previous research relating avian traits to horizontal structure (Sweeney et al., 2025) and the benefits of habitat complementarity (Jones & Tingley, 2022; Ulyshen et al., 2023), we found evidence further supporting the conclusion that horizontally structured forests benefit some bird species. Habitat complementarity may also explain the strong association between high nesting birds and horizontally structured forests. Presence of open habitat is reflected in high values of SD FHD in our analysis, so the strong association between upper canopy nesters and horizontal structure is likely due to some species' preferences for a mosaic of forest types, both open and closed. Some canopy nesting birds like the Northern Parula (*S. americana*) preferentially locate nests near open habitat (Moldenhauer & Regelski, 2020), and other species such as the Pine Warbler (*S. pinus*)—another upper canopy nesting species typically associated with dense coniferous forests—sometimes demonstrate an attraction to edgier habitat in the southern part of their range (Rodewald et al., 2020).

### 4.4 Patterns across vertical and horizontal structure

Mass showed a consistent weakening interaction with relationships to both vertical and horizontal structure, implying that larger bodied birds were less responsive to forest structure in our analysis. This lack of sensitivity to structure might be due to the tendency of larger bodied birds to inhabit larger ranges (Schoener, 1968) and make more generalist use of habitat (Brown, 2007). For example, Wild Turkeys (*M. gallopavo*), the largest bird species in our analysis, use various forest types and have home ranges up to 683 ha in size (Brown, 1980). Larger bodied birds are often better able to persist in seasonal, cold and challenging environments (Keyser et al., 2023; Meiri & Dayan, 2003) and should thus be less sensitive to variation in habitat structure compared to smaller bodied birds. Furthermore, populations of larger birds may only respond to forest structure at broader spatial scales than we tested. Although we assessed structure relationships across 1.5 km radius buffers—which should have captured structure variation relevant to most species (Jackson & Fahrig, 2012; Sherry, 1979)—large birds likely rely on forest structure across much larger extents due to their larger home ranges and dispersal distances (Schoener, 1968).

### 4.5 Limitations

We limited our analysis to a small suite of traits and only two structure metrics to focus on our a priori predictions and to minimize multicollinearity in our model. This narrow focus facilitated strong inference from our model but may have missed other important relationships between avian traits and forest structure. We did not implement beak morphology traits, for example, to limit multicollinearity with diet traits, but such traits may strongly relate to vegetation structure. Furthermore, the three trait databases used in our analysis—EltonTraits (Wilman et al., 2014), AVONET (Tobias et al., 2022) and the Sheard et al. (2024) nest traits database—are subject to uncertainty and unknown biases. Easily discovered nests like ground nests, for instance, are likely better represented in the nest traits database compared to canopy nests. Although each database provides some quantification of uncertainty or intraspecific variation in traits, underlying biases are unknowable with current data. Despite these biases, the trait databases compiled in our analysis represent the current gold standard for implementing trait information across many species and were therefore well suited to our approach. Furthermore, GEDI data products include individual metrics to quantify vegetation density across different vertical height strata, which some bird species may rely on more than the overall vertical distribution of vegetation (FHD) metrics that we focused on our analysis. We chose not to include additional metrics in our analysis because FHD was accurately predicted in our fine‐grained gridded product (Wei et al., 2026) and accomplished our goal of summarizing the entire vertical distribution of the forest canopy, but future work may benefit by relating specific functional traits to individual layers of the forest canopy. There also remains opportunity to test additional trait associations across a larger suite of traits and forest structure metrics by implementing non‐parametric methods that are less susceptible to multicollinearity issues (e.g. random forest), though inferences derived from such work may be weaker than those presented here. Finally, we chose to assess trait associations with forest structure responses at the population level. This decision allowed us to implement eBird data in our analysis to detect signals of trait interactions across the extent of BCR28, but future work may benefit from approaches that model trait interactions at even finer spatial resolutions relevant to individual birds. Although we acknowledge the limits of our approach, we are unaware of existing work explicitly assessing trait interactions with avian responses to vertical and horizontal structure concurrently and at regional scales.

## 5 CONCLUSIONS

In summary, we found that incorporating vertical and horizontal forest structure was important for quantifying species–environment associations for forest‐dependent birds and that these associations could be partially explained by functional traits. Larger bodied birds were less sensitive to both forest structure components, while birds with specialized foraging strata were especially sensitive to vertical forest structure. The use of novel forest structure data and participatory science to assess structure relationships and trait interactions across large spatial extents offers a new window of investigation into species–environment relationships, clarifying the roles of traits and habitat structure across avian communities.

## AUTHOR CONTRIBUTIONS

All authors conceived the ideas; Trevor Roberts and Benjamin Zuckerberg designed methodology; Trevor Roberts analysed the data; Trevor Roberts and Benjamin Zuckerberg led the writing of the manuscript. All authors contributed critically to the drafts and gave final approval for publication.

## CONFLICT OF INTEREST STATEMENT

The authors declare that they have no known competing financial interests or personal relationships that influenced the work reported in this paper.

## Supporting information


**Table S1:** List of predictors used in the occurrence submodel of our two‐level hierarchical Bayesian model.
**Table S2:** Full list of sources used by the clootl R package to construct the pruned phylogenetic tree used in this analysis. The ‘Contribution (%)’ column indicates the percentage of nodes in the tree to which each source contributed; therefore, the column does not sum to 100%.
**Figure S1:** Functional trait distributions used in analysis. HWI and Mass were natural log transformed prior to analysis.
**Figure S2:** Variance explained for each predictor of occurrence in the two‐level hierarchical Bayesian model. Differences in ‘Baseline Occurrence’ were captured with species‐level intercepts.
**Figure S3:** Two‐level hierarchical Bayesian model with phylogenetic and spatial terms included. This model is identical to the model used in our analysis, but with a spatial Gaussian process term (spatial random effect) and phylogenetic correlation matrix (∑_phylo_) included to account for spatial and phylogenetic autocorrelation. This model failed to converge despite sampling posteriors across 200,000 iterations. The model consisted of two levels. The first level (Occurrence Model) fit environmental (Bs), effort (As) and latent factors (Us × Ws) against each species occurrence probability on the logit scale. The second level (Trait Mediation Sub‐model) fitted the B estimates from the first level of the model against species‐level trait values (Γ). Estimated parameters were given uninformative priors. Note the variance element of normal priors is given as precision (1/*σ* ^2^), not standard deviation.
**Figure S4:** Distributions of *p*‐values from Blomberg's *K* test of phylogenetic independence for species responses to horizontal and vertical structure. Species in negative, neutral and positive response groups were tested. The dashed red line indicates *p*‐value = 0.05. *p*‐value distributions indicate no or minimal signal of phylogenetic autocorrelation in all response groups.
**Figure S5:** Semi‐variogram (a) and Moran's *I* results in four distance bins (b) of the residuals of the two‐level hierarchical Bayesian model. Although some spatial autocorrelation was present at small distances, the magnitude of autocorrelation remained very low. Furthermore, posterior Moran's *I* values indicated that spatial autocorrelation was not statistically significant at any scale tested. X‐axes of (a) and (b) are in metres.
**Table S3:** Species‐level relationships to each environmental predictor for all 106 species present during the 2023 breeding season in Bird Conservation Region 28. All coefficients are *z*‐scored ± 95% credible interval. Asterisks indicate no overlap of 95% CI with zero.
**Table S4:** Trait associations with the vertical and horizontal structure relationships of breeding season birds in Bird Conservation Region 28. (All) indicates associations across all species in the community. (+) indicates associations for birds that respond positively to vertical or horizontal structure. (−) indicates associations for birds that respond negatively to vertical or horizontal structure. Bolding and asterisks indicate no overlap between 95% CI and zero.
**Figure S6:** Responses of each of the 106 bird species analysed to vertical forest structure (median FHD) and horizontal forest structure (SD FHD). Species labelled in blue have responses in 80th percentile or above to either horizontal or vertical forest structure. Species with less extreme relationships to forest structure are in grey.
**Figure S7:** Difference in occurrence probability (Pr(Occ)) predictions on the probability scale (0 to 1) between models with and without structure predictors at regional (a; across BCR28) and finer (b; <50 km) scales. Predictions are for Northern parula (*S. americana*). Green pixels (no difference) indicate areas in which structure predictors explain little variation in occurrence. Red pixels (large difference, positive or negative) indicate areas where structure explains substantial variation in occurrence. Although structure predictors have minimal impact on occurrence predictions at regional scale (a), they exert much greater influence at finer scales (b).
**Figure S8:** Distribution of the importance of vertical structure across the entire avian community (106 species) of BCR28. Although the median importance of the vertical structure predictor (median FHD) was only 1.6%, some species (e.g. Northern Parula) experienced much higher importance, reflecting the nuanced importance of structure in our analysis.

## Data Availability

All associated code and intermediate data products relevant to this analysis are available in a Zenodo repository: https://doi.org/10.5281/zenodo.17408555 (Roberts et al., 2026).
