# Supplementary material for: Functional traits explain responses of Appalachian birds to vertical and horizontal forest structure
Source: J Anim Ecol. 2026 Jul 31;95(9):1598–615. doi: 10.1111/1365-2656.70326 (PMC13536234; doi:10.1111/1365-2656.70326)
Supplement: Supplementary file 1 [file JANE-95-1598-s001.docx]

SUPPORTING INFORMATION

List of Predictors

Table S1. List of predictors used in the occurrence submodel of our two-level hierarchical Bayesian model.

| **Predictor** | **Type** | **Source** | **Meaning** |
| --- | --- | --- | --- |
| **Vertical Structure** | Environmental (Structure) | GEDI | Measure of vertical layering of vegetation in 1.5km buffer around eBird checklist; based on median of FHD values |
| **Horizontal Structure** | Environmental (Structure) | GEDI | Measure of horizontal variation in vegetation layering in 1.5km buffer around eBird checklist; based on standard deviation of FHD values |
| **Deciduous** | Environmental | NLCD | Measure of NLCD pixels classified as deciduous in 1.5km buffer around eBird checklist scaled from 0 (no deciduous) to 1 (all deciduous) |
| **Temperature** | Environmental | Daymet | Average annual temperature for 2023 of 1km pixel in which eBird checklist lies |
| **Precipitation** | Environmental | Daymet | Total annual precipitation for 2023 of 1km pixel in which eBird checklist lies |
| **Elevation** | Environmental | GEDI | Elevation estimate of 1km pixel in which eBird checklist lies |
| **Baseline Occurrence** | Intercept | NA | Species-level intercepts to account for baseline differences in species occurrence |
| **Observation Time** | Effort | eBird | Time of day at which eBird checklist began |
| **Observation Duration** | Effort | eBird | Duration of eBird checklist |
| **Observation Distance** | Effort | eBird | Distance traveled by observers during eBird checklist |
| **Observer Number** | Effort | eBird | Number of observers on eBird checklist |
| **Day of Year** | Effort | eBird | Day of year during which eBird checklist conducted |
| **Latent Factor 1** | Latent Factors | NA | Flexible term to capture remaining variation in model residuals; helps control bias |
| **Latent Factor 2** | Latent Factors | NA | Flexible term to capture remaining variation in model residuals; helps control bias |

Phylogenetic Tree Citations

Table S2. Full list of sources used by the clootl R package to construct the pruned phylogenetic tree used in this analysis. The “Contribution (%)” column indicates the percentage of nodes in the tree to which each source contributed, therefore the column does not sum to 100%.

| **Reference** | **DOI** | **Contribution (%)** |
| --- | --- | --- |
| Burleigh, J. Gordon, Rebecca T. Kimball, Edward L. Braun. 2015. Building the avian tree of life using a large-scale, sparse supermatrix. Molecular Phylogenetics and Evolution 84: 53-63 | http://dx.doi.org/10.1016/j.ympev.2014.12.003 | 52.38 |
| Jetz, W., G. H. Thomas, J. B. Joy, K. Hartmann, A. O. Mooers. 2012. The global diversity of birds in space and time. Nature 491 (7424): 444-448 | http://dx.doi.org/10.1038/nature11631 | 51.43 |
| Barker, F. Keith, Kevin J. Burns, John Klicka, Scott M. Lanyon, Irby J. Lovette. 2015. New insights into New World biogeography: An integrated view from the phylogeny of blackbirds, cardinals, sparrows, tanagers, warblers, and allies. The Auk 132 (2): 333-348. | http://dx.doi.org/10.1642/auk-14-110.1 | 31.43 |
| Stiller, J., Feng, S., Chowdhury, AA. et al. Complexity of avian evolution revealed by family-level genomes. Nature (2024). https://doi.org/10.1038/s41586-024-07323-1 | https://doi.org/10.1038/s41586-024-07323-1 | 24.76 |
| Selvatti, Alexandre Pedro, Luiz Pedreira Gonzaga, Claudia Augusta de Moraes Russo. 2015. A Paleogene origin for crown passerines and the diversification of the Oscines in the New World. Molecular Phylogenetics and Evolution 88: 1-15. | http://dx.doi.org/10.1016/j.ympev.2015.03.018 | 22.86 |
| Lovette, Irby J., Jorge L. Pérez-Emán, John P. Sullivan, Richard C. Banks, Isabella Fiorentino, Sergio Córdoba-Córdoba, María Echeverry-Galvis, F. Keith Barker, Kevin J. Burns, John Klicka, Scott M. Lanyon, Eldredge Bermingham. 2010. A comprehensive multilocus phylogeny for the wood-warblers and a revised classification of the Parulidae (Aves). Molecular Phylogenetics and Evolution 57 (2): 753-770. | http://dx.doi.org/10.1016/j.ympev.2010.07.018 | 22.86 |
| Oliveros, C. H., Field, D. J., Ksepka, D. T., Barker, F. K., Aleixo, A., Andersen, M. J., … Faircloth, B. C. (2019). Earth history and the passerine superradiation. Proceedings of the National Academy of Sciences, 116(16), 7916–7925. doi:10.1073/pnas.1813206116 | http://dx.doi.org/10.1073/pnas.1813206116 | 18.10 |
| Kimball, R. T., Oliveros, C. H., Wang, N., White, N. D., Barker, F. K., Field, D. J., … Braun, E. L. (2019). A Phylogenomic Supertree of Birds. Diversity, 11(7), 109. doi:10.3390/d11070109 | http://dx.doi.org/10.3390/d11070109 | 16.19 |
| Reddy, Sushma, Rebecca T. Kimball, Akanksha Pandey, Peter A. Hosner, Michael J. Braun, Shannon J. Hackett, Kin-Lan Han, John Harshman, Christopher J. Huddleston, Sarah Kingston, Ben D. Marks, Kathleen J. Miglia, William S. Moore, Frederick H. Sheldon, Christopher C. Witt, Tamaki Yuri, Edward L. Braun. 2017. Why do phylogenomic data sets yield conflicting trees? Data type influences the avian tree of life more than taxon sampling. Systematic Biology | http://dx.doi.org/10.1093/sysbio/syx041 | 11.43 |
| Claramunt, Santiago, Joel Cracraft. 2015. A new time tree reveals Earth historys imprint on the evolution of modern birds. Science Advances 1 (11): e1501005-e1501005 | http://dx.doi.org/10.1126/sciadv.1501005 | 11.43 |
| Barker, F. K., K. J. Burns, J. Klicka, S. M. Lanyon, I. J. Lovette. 2013. Going to extremes: contrasting rates of diversification in a recent radiation of New World passerine birds. Systematic Biology 62 (2): 298-320. | http://dx.doi.org/10.1093/sysbio/sys094 | 10.48 |
| Price, Trevor D., Daniel M. Hooper, Caitlyn D. Buchanan, Ulf S. Johansson, D. Thomas Tietze, Per Alström, Urban Olsson, Mousumi Ghosh-Harihar, Farah Ishtiaq, Sandeep K. Gupta, Jochen Martens, Bettina Harr, Pratap Singh, Dhananjai Mohan. 2014. Niche filling slows the diversification of Himalayan songbirds. Nature 509: 222-225. | http://dx.doi.org/10.1038/nature13272 | 10.48 |
| Alstrom, P., D. M. Hooper, Y. Liu, U. Olsson, D. Mohan, M. Gelang, H. Le Manh, J. Zhao, F. Lei, T. D. Price. 2014. Discovery of a relict lineage and monotypic family of passerine birds. Biology Letters 10 (3): 20131067-20131067. | http://dx.doi.org/10.1098/rsbl.2013.1067 | 7.62 |
| Wu, S., Rheindt, F. E., Zhang, J., Wang, J., Zhang, L., Quan, C., Li, Z., Wang, M., Wu, F., Qu, Y., Edwards, S. V., Zhou, Z., & Liu, L. (2024). Genomes, fossils, and the concurrent rise of modern birds and flowering plants in the Late Cretaceous. Proceedings of the National Academy of Sciences, 121(8). https://doi.org/10.1073/pnas.2319696121 | http://dx.doi.org/10.1073/pnas.2319696121 | 7.62 |
| Prum, Richard O., Jacob S. Berv, Alex Dornburg, Daniel J. Field, Jeffrey P. Townsend, Emily Moriarty Lemmon, Alan R. Lemmon. 2015. A comprehensive phylogeny of birds (Aves) using targeted next-generation DNA sequencing. Nature 526, (7574): 569-573 | http://dx.doi.org/10.1038/nature15697 | 7.62 |
| Moyle, Robert G., Carl H. Oliveros, Michael J. Andersen, Peter A. Hosner, Brett W. Benz, Joseph D. Manthey, Scott L. Travers, Rafe M. Brown, Brant C. Faircloth. 2016. Tectonic collision and uplift of Wallacea triggered the global songbird radiation. Nature Communications 7: 12709 | http://dx.doi.org/10.1038/ncomms12709 | 7.62 |
| Barker, F. Keith, Alice Cibois, Peter Schikler, Julie Feinstein, Joel Cracraft. 2004. Phylogeny and diversification of the largest avian radiation. Proceedings of the National Academy of Sciences of the United States of America 101 (30): 11040-11045. | http://dx.doi.org/10.1073/pnas.0401892101 | 7.62 |
| Harvey, M. G., Bravo, G. A., Claramunt, S., Cuervo, A. M., Derryberry, G. E., Battilana, J., … Derryberry, E. P. (2020). The evolution of a tropical biodiversity hotspot. Science, 370(6522), 1343–1348. doi:10.1126/science.aaz6970 | http://dx.doi.org/10.1126/science.aaz6970 | 6.67 |
| Päckert, M., Favre, A., Schnitzler, J., Martens, J., Sun, Y., Tietze, D. T., Hailer, F., Michalak, I., & Strutzenberger, P. (2020). “Into and Out of” the Qinghai‐Tibet Plateau and the Himalayas: Centers of origin and diversification across five clades of Eurasian montane and alpine passerine birds. Ecology and Evolution, 10(17), 9283–9300. Portico. https://doi.org/10.1002/ece3.6615 | http://dx.doi.org/10.1002/ece3.6615 | 6.67 |
| Odeen, A., Hastad O., & Alstrom P. 2011. Evolution of ultraviolet vision in the largest avian radiation - the passerines. BMC Evolutionary Biology 11 (313): 1-8. | http://dx.doi.org/10.1186/1471-2148-11-313 | 6.67 |
| McCullough, J. M., Oliveros, C. H., Benz, B. W., Zenil-Ferguson, R., Cracraft, J., Moyle, R. G., & Andersen, M. J. (2022). Wallacean and Melanesian Islands Promote Higher Rates of Diversification within the Global Passerine Radiation Corvides. Systematic Biology. https://doi.org/10.1093/sysbio/syac044 | http://dx.doi.org/10.1093/sysbio/syac044 | 5.71 |
| Barker, F.K. 2014. Mitogenomic data resolve basal relationships among passeriform and passeridan birds. Molecular Phylogenetics and Evolution 79: 313-324. | http://dx.doi.org/10.1016/j.ympev.2014.06.011 | 5.71 |
| Shakya, S. B., Fuchs, J., Pons, J.-M., & Sheldon, F. H. (2017). Tapping the woodpecker tree for evolutionary insight. Molecular Phylogenetics and Evolution, 116, 182–191. doi:10.1016/j.ympev.2017.09.005 | http://dx.doi.org/10.1016/j.ympev.2017.09.005 | 4.76 |
| Dufort, Matthew J. 2015. An augmented supermatrix phylogeny of the avian family Picidae reveals uncertainty deep in the family tree. Molecular Phylogenetics and Evolution | http://dx.doi.org/10.1016/j.ympev.2015.08.025 | 4.76 |
| Ericson, Per GP, Seraina Klopfstein, Martin Irestedt, Jacqueline MT Nguyen and Johan AA Nylander. 2014. Dating the diversification of the major lineages of Passeriformes (Aves). BMC Evolutionary Biology, 14(8). | http://dx.doi.org/10.1186/1471-2148-14-8 | 4.76 |
| Beresford, P., F.K. Barker, P.G. Ryan, T.M. Crowe. 2005. African endemics span the tree of songbirds (Passeri): molecular systematics of several evolutionary 'enigmas'. Proceedings of the Royal Society B: Biological Sciences 272 (1565): 849-858 | http://dx.doi.org/10.1098/rspb.2004.2997 | 4.76 |
| Benz, Brett W., Mark B. Robbins, A. Townsend Peterson. 2006. Evolutionary history of woodpeckers and allies (Aves: Picidae): placing key taxa on the phylogenetic tree. Molecular Phylogenetics and Evolution 40 (2): 389-399. | http://dx.doi.org/10.1016/j.ympev.2006.02.021 | 3.81 |
| Catanach, T. A., Halley, M. R., & Pirro, S. (2024). Enigmas no longer: using ultraconserved elements to place several unusual hawk taxa and address the non-monophyly of the genusAccipiter(Accipitriformes: Accipitridae). Biological Journal of the Linnean Society. https://doi.org/10.1093/biolinnean/blae028 | https://doi.org/10.1093/biolinnean/blae028 | 3.81 |
| Jønsson, Knud Andreas, Pierre-Henri Fabre, Jonathan D. Kennedy, Ben G. Holt, Michael K. Borregaard, Carsten Rahbek, Jon Fjeldså. 2016. A supermatrix phylogeny of corvoid passerine birds (Aves: Corvides). Molecular Phylogenetics and Evolution 94: 87-94. | http://dx.doi.org/10.1016/j.ympev.2015.08.020 | 3.81 |
| Fuchs, Jérôme, Jan I. Ohlson, Per G. P. Ericson, Eric Pasquet. 2007. Synchronous intercontinental splits between assemblages of woodpeckers suggested by molecular data. Zoologica Scripta 36 (1): 11-25 | http://dx.doi.org/10.1111/j.1463-6409.2006.00267.x | 3.81 |
| Simone Treplin, Romy Siegert, Christoph Bleidorn, Hazell Shokellu Thompson, Roger Fotso, Ralph Tiedemann, 2008, 'Molecular phylogeny of songbirds (Aves: Passeriformes) and the relative utility of common nuclear marker loci', Cladistics, vol. 24, no. 3, pp. 328-349 | http://dx.doi.org/10.1111/j.1096-0031.2007.00178.x | 3.81 |
| Zuccon, Dario, Robert Prŷs-Jones, Pamela C. Rasmussen, and Per G.P. Ericson. 2012. The phylogenetic relationships and generic limits of finches (Fringillidae). Molecular Phylogenetics and Evolution 62 (2): 581-596. | http://dx.doi.org/10.1016/j.ympev.2011.10.002 | 3.81 |
| Kennedy, J. D., Marki, P. Z., Reeve, A. H., Blom, M. P. K., Prawiradilaga, D. M., Haryoko, T., Koane, B., Kamminga, P., Irestedt, M., Jønsson, K. A., & Sheard, C. (2022). Diversification and community assembly of the world’s largest tropical island. Global Ecology and Biogeography. Portico. https://doi.org/10.1111/geb.13484 | http://dx.doi.org/10.1111/geb.13484 | 2.86 |
| Salter, J. F., Oliveros, C. H., Hosner, P. A., Manthey, J. D., Robbins, M. B., Moyle, R. G., Brumfield, R. T., & Faircloth, B. C. (2020). Extensive paraphyly in the typical owl family (Strigidae). The Auk, 137(1). https://doi.org/10.1093/auk/ukz070 | http://dx.doi.org/10.1093/auk/ukz070 | 2.86 |
| Gibb, G. C., & Shepherd, L. D. (2022). Recent evolution of extreme sexual dimorphism in the huia (Heteralocha acutirostris; Callaeidae). Molecular Phylogenetics and Evolution, 175, 107575. https://doi.org/10.1016/j.ympev.2022.107575 | http://dx.doi.org/10.1016/j.ympev.2022.107575 | 2.86 |
| Gibb, Gillian C., Ryan England, Gerrit Hartig, P.A. (Trish) McLenachan, Briar L. Taylor Smith, Bennet J. McComish, Alan Cooper, David Penny. 2015. New Zealand passerines help clarify the diversification of major songbird lineages during the Oligocene. Genome Biology and Evolution 7 (11): 2983-2995. | http://dx.doi.org/10.1093/gbe/evv196 | 2.86 |
| Slager, David L., C.J. Battey, Robert W. Bryson, Gary Voelker, John Klicka. 2014. A multilocus phylogeny of a major New World avian radiation: The Vireonidae. Molecular Phylogenetics and Evolution 80: 95-104 | http://dx.doi.org/10.1016/j.ympev.2014.07.021 | 2.86 |
| Ulf S. Johansson, Jon Fjeldså, Rauri C.K. Bowie, 2008, 'Phylogenetic relationships within Passerida (Aves: Passeriformes): A review and a new molecular phylogeny based on three nuclear intron markers', Molecular Phylogenetics and Evolution, vol. 48, no. 3, pp. 858-876 | http://dx.doi.org/10.1016/j.ympev.2008.05.029 | 2.86 |
| Hugall, Andrew F., Devi Stuart-Fox. 2012. Accelerated speciation in colour-polymorphic birds. Nature 485 (7400): 631-634. | http://dx.doi.org/10.1038/nature11050 | 1.90 |
| eBird Taxonomy draft, 2021, 2023 | https://ebird.org/science/use-ebird-data/the-ebird-taxonomy | 1.90 |
| Cicero, C., Mason, N. A., Benedict, L., & Rising, J. D. (2020). Behavioral, morphological, and ecological trait evolution in two clades of New World Sparrows (AimophilaandPeucaea, Passerellidae). PeerJ, 8, e9249. Portico. https://doi.org/10.7717/peerj.9249 | http://dx.doi.org/10.7717/peerj.9249 | 1.90 |
| Zhao, M., Gordon Burleigh, J., Olsson, U., Alström, P., & Kimball, R. T. (2023). A near-complete and time-calibrated phylogeny of the Old World flycatchers, robins and chats (Aves, Muscicapidae). Molecular Phylogenetics and Evolution, 178, 107646. https://doi.org/10.1016/j.ympev.2022.107646 | http://dx.doi.org/10.1016/j.ympev.2022.107646 | 1.90 |
| Wood, Jamie R., Kieren J. Mitchell, R. Paul Scofield, Vanesa L. De Pietri, Nicolas J. Rawlence, Alan Cooper. 2016. Phylogenetic relationships and terrestrial adaptations of the extinct laughing owl, Sceloglaux albifacies (Aves: Strigidae). Zoological Journal of the Linnean Society | http://dx.doi.org/10.1111/zoj.12483 | 1.90 |
| Lerner, H., M. Klaver, D. Mindell. 2008. Molecular phylogenetics of the Buteonine birds of prey (Accipitridae). The Auk 125 (2): 304-315. | http://dx.doi.org/10.1525/auk.2008.06161 | 1.90 |
| Powell, Alexis F.L.A., F. Keith Barker, Scott M. Lanyon, Kevin J. Burns, John Klicka, Irby J. Lovette. 2014. A comprehensive species-level molecular phylogeny of the New World blackbirds (Icteridae). Molecular Phylogenetics and Evolution 71: 94-112. | http://dx.doi.org/10.1016/j.ympev.2013.11.009 | 1.90 |
| Pasquet, Eric, F. Keith Barker, Jochen Martens, Annie Tillier, Corinne Cruaud, Alice Cibois. 2014. Evolution within the nuthatches (Sittidae: Aves, Passeriformes): molecular phylogeny, biogeography, and ecological perspectives. Journal of Ornithology 155 (3):755-765. | http://dx.doi.org/10.1007/s10336-014-1063-7 | 1.90 |
| Hosner, Peter A. Brant C. Faircloth, Travis C. Glenn, Edward L. Braun, Rebecca T. Kimball. 2015. Avoiding missing data biases in phylogenomic inference: an empirical study in the landfowl (Aves: Galliformes). Molecular Biology and Evolution 33 (4): 1110-1125 | http://dx.doi.org/10.1093/molbev/msv347 | 0.95 |
| Payne, Robert B. The cuckoos. Vol. 15. Oxford University Press, 2005.  ISBN-13: 978-0198502135 ISBN-10: 0198502133 | http://ukcatalogue.oup.com/product/9780198502135.do | 0.95 |
| Harris, R.B., M.D. Carling, I.J. Lovette. 2014. The influence of sampling design on species tree inference: a new relationship for the New World chickadees (Aves: Poecile). Evolution 68 (2): 501-513. | http://dx.doi.org/10.1111/evo.12280 | 0.95 |
| Chen, D., Hosner, P. A., Dittmann, D. L., O’Neill, J. P., Birks, S. M., Braun, E. L., & Kimball, R. T. (2021). Divergence time estimation of Galliformes based on the best gene shopping scheme of ultraconserved elements. BMC Ecology and Evolution, 21(1). https://doi.org/10.1186/s12862-021-01935-1 | http://dx.doi.org/10.1186/s12862-021-01935-1 | 0.95 |
| Imfeld, Barker, Brumfield, Mitochondrial genomes and thousands of ultraconserved elements resolve the taxonomy and historical biogeography of the Euphonia and Chlorophonia finches (Passeriformes: Fringillidae), The Auk, Volume 137, Issue 3, 1 July 2020, ukaa016, | https://doi.org/10.1093/auk/ukaa016 | 0.95 |
| Yonezawa, T., Segawa, T., Mori, H., Campos, P. F., Hongoh, Y., Endo, H., Akiyoshi, A., Kohno, N., Nishida, S., Wu, J., Jin, H., Adachi, J., Kishino, H., Kurokawa, K., Nogi, Y., Tanabe, H., Mukoyama, H., Yoshida, K., Rasoamiaramanana, A., … Hasegawa, M. (2017). Phylogenomics and Morphology of Extinct Paleognaths Reveal the Origin and Evolution of the Ratites. Current Biology, 27(1), 68–77. https://doi.org/10.1016/j.cub.2016.10.029 | http://dx.doi.org/10.1016/j.cub.2016.10.029 | 0.95 |
| Bryson, R. W., Faircloth, B. C., Tsai, W. L. E., McCormack, J. E., & Klicka, J. (2016). Target enrichment of thousands of ultraconserved elements sheds new light on early relationships within New World sparrows (Aves: Passerellidae). The Auk, 133(3), 451–458. https://doi.org/10.1642/auk-16-26.1 | http://dx.doi.org/10.1642/auk-16-26.1 | 0.95 |
| Oliver, P. M., Heiniger, H., Hugall, A. F., Joseph, L., & Mitchell, K. J. (2020). Oligocene divergence of frogmouth birds (Podargidae) across Wallace’s Line. Biology Letters, 16(5), 20200040. https://doi.org/10.1098/rsbl.2020.0040 | http://dx.doi.org/10.1098/rsbl.2020.0040 | 0.95 |
| Hosner, P. A., Braun, E. L., & Kimball, R. T. (2016). Rapid and recent diversification of curassows, guans, and chachalacas (Galliformes: Cracidae) out of Mesoamerica: Phylogeny inferred from mitochondrial, intron, and ultraconserved element sequences. Molecular Phylogenetics and Evolution, 102, 320–330. https://doi.org/10.1016/j.ympev.2016.06.006 | http://dx.doi.org/10.1016/j.ympev.2016.06.006 | 0.95 |
| Nagy, J., & Tökölyi, J. (2014). Phylogeny, Historical Biogeography and the Evolution of Migration in Accipitrid Birds of Prey (Aves: Accipitriformes). Ornis Hungarica, 22(1), 15–35. https://doi.org/10.2478/orhu-2014-0008 | http://dx.doi.org/10.2478/orhu-2014-0008 | 0.95 |
| Vázquez-López, M., Ramírez-Barrera, S. M., Terrones-Ramírez, A. K., Robles-Bello, S. M., Nieto-Montes de Oca, A., Ruegg, K., & Hernández-Baños, B. E. (2024). Biogeographic factors contributing to the diversification of Euphoniinae (Aves, Passeriformes, Fringillidae): a phylogenetic and ancestral areas analysis. ZooKeys, 1188, 169–195. https://doi.org/10.3897/zookeys.1188.107047 | https://doi.org/10.3897/zookeys.1188.107047 | 0.95 |
| Stein, R. Will, Joseph W. Brown, Arne Ø. Mooers. 2015. A molecular genetic time scale demonstrates Cretaceous origins and multiple diversification rate shifts within the order Galliformes (Aves). Molecular Phylogenetics and Evolution 92: 155-164 | http://dx.doi.org/10.1016/j.ympev.2015.06.005 | 0.95 |
| Wang, Ning, Rebecca T. Kimball, Edward L. Braun, Bin Liang, Zhengwang Zhang. 2016. Ancestral range reconstruction of Galliformes: the effects of topology and taxon sampling. Journal of Biogeography | http://dx.doi.org/10.1111/jbi.12782 | 0.95 |
| Robert G. Moyle, Joel Cracraft, Maklarin Lakim, Jamili Nais, Frederick H. Sheldon, 2006, 'Reconsideration of the phylogenetic relationships of the enigmatic Bornean Bristlehead (Pityriasis gymnocephala)', Molecular Phylogenetics and Evolution, vol. 39, no. 3, pp. 893-898 | http://dx.doi.org/10.1016/j.ympev.2006.01.024 | 0.95 |
| Rheindt, Frank E., Janette A. Norman, Les Christidis. 2008. Phylogenetic relationships of tyrant-flycatchers (Aves: Tyrannidae), with an emphasis on the elaeniine assemblage. Molecular Phylogenetics and Evolution 46 (1): 88-101 | http://dx.doi.org/10.1016/j.ympev.2007.09.011 | 0.95 |
| Scofield, R. Paul, Kieren J. Mitchell, Jamie R. Wood, Vanesa L. De Pietri, Scott Jarvie, Bastien Llamas, Alan Cooper. 2016. The Origin and Phylogenetic Relationships of the New Zealand Ravens. Molecular Phylogenetics and Evolution 106: 136–143 | http://dx.doi.org/10.1016/j.ympev.2016.09.022 | 0.95 |
| Jérôme Fuchs, Jean-Marc Pons, Steven M Goodman, Vincent Bretagnolle, Martim Melo, Rauri CK Bowie, David Currie, Roger Safford, Munir Z Virani, Simon Thomsett, Alawi Hija, Corinne Cruaud, Eric Pasquet, 2008, 'Tracing the colonization history of the Indian Ocean scops-owls (Strigiformes: Otus) with further insight into the spatio-temporal origin of the Malagasy avifauna', BMC Evolutionary Biology, vol. 8, no. 1, p. 197 | http://dx.doi.org/10.1186/1471-2148-8-197 | 0.95 |
| Mann, Nigel I., F. Keith Barker, Jeff A. Graves, Kimberly A. Dingess-Mann, Peter J.B. Slater. 2006. Molecular data delineate four genera of “Thryothorus” wrens. Molecular Phylogenetics and Evolution 40 (3): 750-759 | http://dx.doi.org/10.1016/j.ympev.2006.04.014 | 0.95 |
| Kimball, Rebecca T., Colette M. St. Mary, Edward L. Braun. 2011. A macroevolutionary perspective on multiple sexual traits in the Phasianidae (Galliformes). International Journal of Evolutionary Biology 2011: 1-16. | http://dx.doi.org/10.4061/2011/423938 | 0.95 |
| Han, Kin-Lan, Mark B. Robbins, Michael J. Braun. 2010. A multi-gene estimate of phylogeny in the nightjars and nighthawks (Caprimulgidae). Molecular Phylogenetics and Evolution 55 (2): 443-453. | http://dx.doi.org/10.1016/j.ympev.2010.01.023 | 0.95 |
| Klicka, John, F. Keith Barker, Kevin J. Burns, Scott M. Lanyon, Irby J. Lovette, Jaime A. Chaves, Robert W. Bryson. 2014. A comprehensive multilocus assessment of sparrow (Aves: Passerellidae) relationships. Molecular Phylogenetics and Evolution 77: 177–182. | http://dx.doi.org/10.1016/j.ympev.2014.04.025 | 0.95 |

Trait Distributions

Figure S1. Functional trait distributions used in analysis. HWI and Mass were natural log transformed prior to analysis.

Variable Importance

Figure S2. Variance explained for each predictor of occurrence in the two-level hierarchical Bayesian model. Differences in “Baseline Occurrence” were captured with species-level intercepts.

Two-Level Hierarchical Bayesian Model with Phylogenetic and Spatial Terms

Figure S3. Two-level hierarchical Bayesian model with phylogenetic and spatial terms included. This model is identical to the model used in our analysis, but with a spatial gaussian process term (spatial random effect) and phylogenetic correlation matrix (∑_phylo_) included to account for spatial and phylogenetic autocorrelation. This model failed to converge despite sampling posteriors across 200,000 iterations. The model consisted of two levels. The first level (Occurrence Model) fit environmental (Bs), effort (As), and latent factors (Us*Ws) against each species occurrence probability on the logit scale. The second level (Trait Mediation Sub-model) fitted the B estimates from the first level of the model against species-level trait values (Γ). Estimated parameters were given uninformative priors. Note the variance element of normal priors is given as precision (1 / σ^2^), not standard deviation.

Model Diagnostics

Several diagnostics of the two-level Bayesian hierarchical model provided confidence in estimates of species’ occurrence responses to forest structure. For example, we detected no phylogenetic signal in species' responses to environmental predictors. The lack of phylogenetic structure was supported by the failure of convergence for the phylogenetic version of the two-level Bayesian hierarchical model (even after 200,000 iterations), and also by the insignificant phylogenetic structure in model residuals (λ ≈ 0; p = 1) or species’ response coefficients (Figure S4). The trait data may have sufficiently captured phylogenetic relationships between species, negating the need for an explicit phylogenetic autocorrelation term. In short, model diagnostics indicated that little to no phylogenetic autocorrelation was present between observations, so we continued our analysis using the base model without an explicit phylogenetic term.

Diagnostics also indicated negligible impact of spatial autocorrelation on our results. A residual semi-variogram demonstrated little change in semi-variance between residuals at spatial scales below 10 km and residuals at coarser scales (Figure S5). Similarly, Moran’s I tests of spatial autocorrelation were insignificant at all tested spatial scales (Figure S5). Because we detected no spatial autocorrelation in our results, we opted not to include a spatial autocorrelation term in the model to limit model complexity and computational burden.

We used a latent factor term to flexibly capture leftover residual variation across species and checklists, but latent factors can sometimes diminish identification of other, significant model predictors (Doser et al., 2023). To ensure that the latent factors did not map onto the same variation explained by environmental and effort predictors, we assessed correlations between model posteriors. We found no indication of strong correlations between posteriors (0.001 < r < 0.008), increasing confidence in the interpretability of our results.

Phylogenetic Autocorrelation

Figure S4. Distributions of p-values from Blomberg’s K test of phylogenetic independence for species responses to horizontal and vertical structure. Species in negative, neutral, and positive response groups were tested. The dashed red line indicates p-value = 0.05. P-value distributions indicate no or minimal signal of phylogenetic autocorrelation in all response groups.

Spatial Autocorrelation

Figure S5. Semi-variogram (a) and Moran’s I results in four distance bins (b) of the residuals of the two-level hierarchical Bayesian model. Although some spatial autocorrelation was present at small distances, the magnitude of autocorrelation remained very low. Furthermore, posterior Moran’s I values indicated that spatial autocorrelation was not statistically significant at any scale tested. X-axes of (a) and (b) are in meters.

Species-Environment Relationships

Table S3. Species-level relationships to each environmental predictor for all 106 species present during the 2023 breeding season in Bird Conservation Region 28. All coefficients are z-scored ± 95% credible interval. Asterisks indicate no overlap of 95% CI with zero.

| **Species** | **Family** | **Vertical Str.** | **Horizontal Str.** | **Deciduous** | **Temp** | **Precip** | **Elev** | **Base Occurrence** |
| --- | --- | --- | --- | --- | --- | --- | --- | --- |
| **Sharp-shinned Hawk** | Accipitridae | -0.17 ± 0.32 | -0.07 ± 0.32 | -0.07 ± 0.36 | -0.29 ± 0.45 | -0.06 ± 0.34 | -0.29 ± 0.43 | -6.13 ± 0.54* |
| **Northern Saw-whet Owl** | Strigidae | -0.11 ± 0.44 | 0.16 ± 0.5 | -0.01 ± 0.59 | -0.9 ± 1.15 | 0 ± 0.48 | -0.5 ± 0.96 | -9.88 ± 2.03* |
| **Chuck-will's-widow** | Caprimulgidae | -0.2 ± 0.45 | 0.27 ± 0.42 | 0.12 ± 0.52 | 1.89 ± 0.83* | -0.05 ± 0.48 | -1.13 ± 1.01* | -10.21 ± 2.05* |
| **Eastern Whip-poor-will** | Caprimulgidae | -0.37 ± 0.21* | -0.06 ± 0.19 | 0.16 ± 0.2 | 0.44 ± 0.23* | -0.38 ± 0.23* | -0.64 ± 0.29* | -4.77 ± 0.31* |
| **Ruby-throated Hummingbird** | Trochilidae | -0.02 ± 0.11 | 0.21 ± 0.09* | 0.08 ± 0.1 | -0.01 ± 0.11 | 0.02 ± 0.11 | -0.18 ± 0.11* | -2.55 ± 0.12* |
| **Tufted Titmouse** | Paridae | 0.09 ± 0.09 | 0.14 ± 0.07* | 0.18 ± 0.08* | 0.73 ± 0.09* | 0.02 ± 0.09 | -0.32 ± 0.09* | -0.72 ± 0.07* |
| **Cedar Waxwing** | Bombycillidae | 0.02 ± 0.1 | 0.25 ± 0.08* | 0.1 ± 0.08* | -0.32 ± 0.1* | 0.01 ± 0.1 | -0.15 ± 0.09* | -2.03 ± 0.1* |
| **Great Horned Owl** | Strigidae | -0.03 ± 0.38 | 0.26 ± 0.35 | 0.29 ± 0.42 | 0.3 ± 0.56 | -0.01 ± 0.4 | -0.17 ± 0.55 | -7.21 ± 0.9* |
| **Red-tailed Hawk** | Accipitridae | -0.05 ± 0.16 | 0.36 ± 0.12* | 0.32 ± 0.15* | -0.06 ± 0.17 | -0.1 ± 0.16 | -0.29 ± 0.16* | -3.61 ± 0.18* |
| **Red-shouldered Hawk** | Accipitridae | -0.03 ± 0.15 | 0.17 ± 0.11* | -0.1 ± 0.12 | 0.39 ± 0.14* | -0.06 ± 0.14 | -0.3 ± 0.15* | -3.23 ± 0.16* |
| **Broad-winged Hawk** | Accipitridae | -0.04 ± 0.12 | 0 ± 0.1 | -0.11 ± 0.1* | 0.02 ± 0.12 | 0.14 ± 0.12* | -0.02 ± 0.11 | -2.74 ± 0.12* |
| **Canada Warbler** | Parulidae | -0.12 ± 0.13 | -0.4 ± 0.14* | 0.04 ± 0.12 | -0.81 ± 0.18* | -0.17 ± 0.13* | 1 ± 0.13* | -3.82 ± 0.23* |
| **Wilson's Warbler** | Parulidae | 0.06 ± 0.4 | -0.07 ± 0.42 | -0.21 ± 0.46 | -0.46 ± 0.72 | -0.02 ± 0.42 | -0.34 ± 0.65 | -8.51 ± 1.37* |
| **Bicknell's Thrush** | Turdidae | -0.16 ± 0.48 | -0.19 ± 0.54 | -0.76 ± 0.61* | -2.7 ± 1.26* | 0.33 ± 0.51 | 0 ± 0.89 | -12.73 ± 3.01* |
| **Veery** | Turdidae | -0.08 ± 0.09 | -0.19 ± 0.09* | 0.2 ± 0.09* | -1.01 ± 0.12* | 0.17 ± 0.09* | 0.32 ± 0.08* | -2.21 ± 0.11* |
| **Hermit Thrush** | Turdidae | 0.1 ± 0.12 | -0.32 ± 0.13* | -0.29 ± 0.11* | -1.62 ± 0.18* | -0.16 ± 0.13* | 0.2 ± 0.12* | -3.81 ± 0.22* |
| **Gray-cheeked Thrush** | Turdidae | -0.01 ± 0.4 | 0.06 ± 0.41 | 0.02 ± 0.51 | 0 ± 0.75 | -0.08 ± 0.44 | -1.2 ± 0.86* | -8.54 ± 1.36* |
| **Swainson's Thrush** | Turdidae | 0.13 ± 0.18 | -0.17 ± 0.15* | -0.12 ± 0.15 | -0.21 ± 0.18* | 0.06 ± 0.17 | -0.55 ± 0.2* | -4.07 ± 0.23* |
| **Brown Creeper** | Certhiidae | 0.24 ± 0.17* | -0.36 ± 0.17* | -0.58 ± 0.15* | -1.24 ± 0.22* | -0.19 ± 0.15* | 0.46 ± 0.15* | -4.44 ± 0.28* |
| **Evening Grosbeak** | Fringillidae | 0.03 ± 0.45 | 0.41 ± 0.49 | -0.2 ± 0.62 | -0.83 ± 1 | -0.31 ± 0.48 | -0.6 ± 0.93 | -9.96 ± 1.8* |
| **Yellow-billed Cuckoo** | Cuculidae | -0.11 ± 0.12 | -0.02 ± 0.1 | 0.29 ± 0.11* | 0.08 ± 0.13 | -0.41 ± 0.13* | -0.82 ± 0.14* | -3.17 ± 0.16* |
| **Black-billed Cuckoo** | Cuculidae | -0.03 ± 0.19 | 0.28 ± 0.16* | 0.36 ± 0.21* | -0.7 ± 0.24* | -0.33 ± 0.21* | -0.53 ± 0.24* | -4.57 ± 0.3* |
| **Olive-sided Flycatcher** | Tyrannidae | -0.04 ± 0.46 | 0.08 ± 0.44 | -0.11 ± 0.54 | -0.33 ± 0.78 | -0.28 ± 0.47 | -0.82 ± 0.89 | -8.08 ± 1.19* |
| **Eastern Wood-Pewee** | Tyrannidae | -0.12 ± 0.09* | -0.03 ± 0.07 | 0.56 ± 0.08* | 0.21 ± 0.09* | -0.38 ± 0.09* | -0.29 ± 0.09* | -1.19 ± 0.08* |
| **Ruby-crowned Kinglet** | Regulidae | -0.13 ± 0.2 | -0.03 ± 0.19 | -0.14 ± 0.2 | -0.98 ± 0.27* | -0.1 ± 0.21 | -0.39 ± 0.23* | -7.94 ± 0.85* |
| **Common Raven** | Corvidae | -0.18 ± 0.1* | -0.08 ± 0.09 | 0.17 ± 0.09* | -0.37 ± 0.11* | -0.42 ± 0.11* | 0.4 ± 0.09* | -2.38 ± 0.11* |
| **Blue Jay** | Corvidae | -0.02 ± 0.08 | 0.12 ± 0.07* | -0.14 ± 0.07* | -0.26 ± 0.08* | -0.03 ± 0.08 | -0.39 ± 0.07* | -0.82 ± 0.07* |
| **Downy Woodpecker** | Picidae | 0 ± 0.1 | 0.07 ± 0.08 | 0.25 ± 0.09* | 0.31 ± 0.1* | 0.04 ± 0.1 | -0.27 ± 0.1* | -2.17 ± 0.11* |
| **Hairy Woodpecker** | Picidae | -0.11 ± 0.11 | -0.06 ± 0.1 | 0.04 ± 0.1 | -0.15 ± 0.11* | -0.09 ± 0.11 | -0.03 ± 0.11 | -2.61 ± 0.12* |
| **Pileated Woodpecker** | Picidae | 0.02 ± 0.1 | -0.04 ± 0.08 | 0.07 ± 0.08 | 0.47 ± 0.09* | -0.22 ± 0.1* | -0.26 ± 0.09* | -1.73 ± 0.09* |
| **Yellow-bellied Flycatcher** | Tyrannidae | 0.15 ± 0.47 | -0.07 ± 0.5 | -0.48 ± 0.63 | -1.92 ± 1.19* | 0.18 ± 0.48 | -0.12 ± 0.87 | -11.27 ± 2.59* |
| **Least Flycatcher** | Tyrannidae | 0.06 ± 0.12 | -0.05 ± 0.12 | 0.41 ± 0.12* | -1.37 ± 0.17* | -0.08 ± 0.13 | 0.26 ± 0.1* | -3.41 ± 0.19* |
| **Acadian Flycatcher** | Tyrannidae | 0.15 ± 0.1* | -0.34 ± 0.09* | 0.18 ± 0.09* | 0.76 ± 0.11* | -0.44 ± 0.1* | -0.29 ± 0.11* | -2.22 ± 0.11* |
| **Merlin** | Falconidae | -0.12 ± 0.43 | 0.28 ± 0.44 | 0.05 ± 0.56 | -0.78 ± 0.87 | -0.26 ± 0.46 | -0.8 ± 0.9 | -8.7 ± 1.45* |
| **Kentucky Warbler** | Parulidae | 0.53 ± 0.19* | 0.16 ± 0.14* | 0.66 ± 0.18* | 1.2 ± 0.21* | -0.29 ± 0.19* | -1.04 ± 0.25* | -5.02 ± 0.32* |
| **Mourning Warbler** | Parulidae | 0.45 ± 0.21* | -0.05 ± 0.22 | 0.3 ± 0.23* | -2.52 ± 0.41* | -0.66 ± 0.26* | 0.28 ± 0.26* | -6.33 ± 0.58* |
| **Purple Finch** | Fringillidae | 0.05 ± 0.2 | 0.19 ± 0.19 | 0.06 ± 0.22 | -2.22 ± 0.37* | -0.28 ± 0.21* | -0.44 ± 0.25* | -5.98 ± 0.53* |
| **Worm-eating Warbler** | Parulidae | 0.12 ± 0.13 | -0.32 ± 0.1* | 0.03 ± 0.09 | 0.95 ± 0.13* | -0.47 ± 0.13* | -0.12 ± 0.13 | -2.93 ± 0.15* |
| **Wood Thrush** | Turdidae | 0.23 ± 0.09* | -0.04 ± 0.07 | 0.3 ± 0.08* | 0.19 ± 0.09* | -0.18 ± 0.08* | -0.33 ± 0.08* | -1.05 ± 0.08* |
| **Yellow-breasted Chat** | Icteriidae | 0.11 ± 0.21 | 0.39 ± 0.15* | 0.4 ± 0.18* | 1.38 ± 0.23* | -0.23 ± 0.21* | -0.49 ± 0.27* | -4.84 ± 0.34* |
| **Baltimore Oriole** | Icteridae | 0.09 ± 0.13 | 0.37 ± 0.1* | 0.21 ± 0.12* | -1.12 ± 0.16* | -0.15 ± 0.13* | -1.61 ± 0.19* | -3.78 ± 0.23* |
| **Orchard Oriole** | Icteridae | -0.01 ± 0.25 | 0.61 ± 0.19* | 0.42 ± 0.24* | 0.66 ± 0.26* | -0.47 ± 0.24* | -0.55 ± 0.3* | -5.9 ± 0.55* |
| **Mississippi Kite** | Accipitridae | 0 ± 0.48 | 0.47 ± 0.51 | 0.58 ± 0.64 | 0.68 ± 0.93 | -0.37 ± 0.5 | -1.86 ± 1.15* | -9.85 ± 1.99* |
| **Dark-eyed Junco** | Passerellidae | -0.07 ± 0.09 | -0.2 ± 0.09* | -0.18 ± 0.08* | -0.98 ± 0.12* | 0.04 ± 0.09 | 1.22 ± 0.1* | -2.21 ± 0.12* |
| **Tennessee Warbler** | Parulidae | 0.49 ± 0.22* | 0.03 ± 0.16 | 0.26 ± 0.19* | -0.33 ± 0.22* | 0.17 ± 0.21 | -1.18 ± 0.27* | -5.66 ± 0.43* |
| **Nashville Warbler** | Parulidae | 0.07 ± 0.24 | -0.11 ± 0.23 | 0.06 ± 0.26 | -1.21 ± 0.36* | -0.16 ± 0.26 | -0.81 ± 0.37* | -6.21 ± 0.59* |
| **Red Crossbill** | Fringillidae | -0.05 ± 0.23 | -0.31 ± 0.27* | -0.46 ± 0.21* | -0.21 ± 0.35 | -0.48 ± 0.27* | 1.34 ± 0.27* | -5.61 ± 0.45* |
| **Eastern Screech-Owl** | Strigidae | -0.09 ± 0.32 | 0.08 ± 0.31 | 0.09 ± 0.35 | 0.7 ± 0.45* | -0.06 ± 0.36 | -0.22 ± 0.49 | -6.21 ± 0.58* |
| **Red-bellied Woodpecker** | Picidae | 0.13 ± 0.11* | 0.26 ± 0.08* | 0.31 ± 0.09* | 0.49 ± 0.1* | -0.13 ± 0.1* | -0.73 ± 0.11* | -1.91 ± 0.11* |
| **Red-headed Woodpecker** | Picidae | -0.06 ± 0.24 | 0.46 ± 0.19* | 0.49 ± 0.26* | 0.72 ± 0.28* | -0.28 ± 0.26* | -0.3 ± 0.31* | -5.3 ± 0.39* |
| **Wild Turkey** | Phasianidae | 0 ± 0.13 | 0.19 ± 0.1* | 0.12 ± 0.11* | 0.29 ± 0.13* | 0.02 ± 0.14 | 0.05 ± 0.12 | -2.88 ± 0.12* |
| **Black-and-white Warbler** | Parulidae | 0.08 ± 0.09 | -0.3 ± 0.07* | 0.14 ± 0.07* | 0.05 ± 0.09 | 0.16 ± 0.08* | -0.1 ± 0.08* | -0.74 ± 0.07* |
| **Brown-headed Cowbird** | Icteridae | -0.07 ± 0.12 | 0.28 ± 0.09* | 0.49 ± 0.11* | 0.04 ± 0.11 | -0.13 ± 0.11* | -0.5 ± 0.11* | -2.63 ± 0.14* |
| **Great Crested Flycatcher** | Tyrannidae | -0.58 ± 0.11* | 0.11 ± 0.09* | 0.34 ± 0.1* | 0.51 ± 0.11* | -0.28 ± 0.1* | -0.86 ± 0.13* | -2.76 ± 0.14* |
| **Louisiana Waterthrush** | Parulidae | 0.21 ± 0.1* | -0.19 ± 0.08* | -0.12 ± 0.08* | 0.27 ± 0.09* | -0.17 ± 0.1* | -0.54 ± 0.11* | -2.1 ± 0.1* |
| **Fox Sparrow** | Passerellidae | 0.04 ± 0.47 | 0.22 ± 0.52 | -0.07 ± 0.65 | -0.11 ± 1.35 | -0.05 ± 0.5 | -0.03 ± 0.99 | -10.37 ± 2.32* |
| **Blue Grosbeak** | Cardinalidae | -0.1 ± 0.32 | 0.74 ± 0.25* | 0.28 ± 0.29 | 1.73 ± 0.42* | -0.01 ± 0.34 | -0.08 ± 0.41 | -7.03 ± 0.77* |
| **Indian Peafowl** | Phasianidae | -0.2 ± 0.51 | 0.26 ± 0.55 | -0.12 ± 0.66 | 0.3 ± 1.27 | -0.11 ± 0.55 | 0.12 ± 1.03 | -10.88 ± 2.51* |
| **Rose-breasted Grosbeak** | Cardinalidae | -0.02 ± 0.1 | 0.05 ± 0.09 | 0.44 ± 0.1* | -0.99 ± 0.13* | -0.04 ± 0.1 | -0.03 ± 0.1 | -2.63 ± 0.14* |
| **Eastern Towhee** | Passerellidae | -0.21 ± 0.08* | 0.08 ± 0.07* | 0.43 ± 0.07* | 0.24 ± 0.08* | -0.36 ± 0.08* | 0.61 ± 0.07* | -0.21 ± 0.06* |
| **Scarlet Tanager** | Cardinalidae | -0.12 ± 0.09* | -0.18 ± 0.08* | 0.44 ± 0.08* | 0.2 ± 0.09* | -0.28 ± 0.09* | -0.41 ± 0.09* | -0.73 ± 0.08* |
| **Summer Tanager** | Cardinalidae | -0.02 ± 0.29 | -0.04 ± 0.22 | 0.46 ± 0.24* | 2.05 ± 0.38* | -0.28 ± 0.31 | -1.22 ± 0.46* | -7.02 ± 0.64* |
| **Black-capped Chickadee** | Paridae | 0 ± 0.11 | 0.02 ± 0.09 | -0.18 ± 0.1* | -2.07 ± 0.16* | -0.33 ± 0.11* | -0.65 ± 0.11* | -3.09 ± 0.17* |
| **Carolina Chickadee** | Paridae | 0.15 ± 0.11* | 0.09 ± 0.08* | 0.08 ± 0.09 | 1.92 ± 0.14* | -0.37 ± 0.11* | 0.75 ± 0.12* | -2.28 ± 0.13* |
| **Blue-gray Gnatcatcher** | Polioptilidae | -0.03 ± 0.11 | 0 ± 0.09 | 0.08 ± 0.09 | 1.02 ± 0.11* | -0.4 ± 0.1* | -0.95 ± 0.12* | -2.35 ± 0.13* |
| **Purple Martin** | Hirundinidae | -0.04 ± 0.34 | 0.42 ± 0.28* | 0.28 ± 0.35 | 0.43 ± 0.37* | 0.09 ± 0.32 | -1.68 ± 0.61* | -7.05 ± 0.86* |
| **Prothonotary Warbler** | Parulidae | 0.01 ± 0.39 | 0.09 ± 0.33 | -0.02 ± 0.38 | 0.75 ± 0.48* | -0.5 ± 0.4* | -1.55 ± 0.76* | -7.65 ± 0.98* |
| **Golden-crowned Kinglet** | Regulidae | -0.21 ± 0.16* | -0.51 ± 0.21* | -0.71 ± 0.15* | -1.13 ± 0.26* | -0.32 ± 0.17* | 1.36 ± 0.17* | -5.26 ± 0.38* |
| **Eastern Phoebe** | Tyrannidae | 0.11 ± 0.09* | 0.14 ± 0.07* | -0.08 ± 0.07* | 0.11 ± 0.08* | 0.06 ± 0.08 | -0.29 ± 0.08* | -1.03 ± 0.07* |
| **American Woodcock** | Scolopacidae | -0.03 ± 0.34 | 0 ± 0.33 | 0.19 ± 0.38 | -0.83 ± 0.53* | -0.27 ± 0.36 | -0.67 ± 0.54* | -6.64 ± 0.72* |
| **Ovenbird** | Parulidae | -0.11 ± 0.1* | -0.44 ± 0.09* | 0.43 ± 0.08* | -0.68 ± 0.1* | -0.18 ± 0.09* | -0.43 ± 0.09* | 0.07 ± 0.08 |
| **Northern Parula** | Parulidae | 0.4 ± 0.1* | -0.13 ± 0.08* | -0.4 ± 0.07* | 0.71 ± 0.09* | -0.06 ± 0.09 | 0.15 ± 0.09* | -1.64 ± 0.09* |
| **Black-throated Blue Warbler** | Parulidae | 0.31 ± 0.12* | -0.39 ± 0.11* | -0.09 ± 0.1 | -1.27 ± 0.15* | 0.31 ± 0.12* | 0.64 ± 0.11* | -2.71 ± 0.18* |
| **Bay-breasted Warbler** | Parulidae | -0.12 ± 0.24 | -0.1 ± 0.21 | 0.03 ± 0.22 | 0.14 ± 0.27 | -0.22 ± 0.25 | -0.79 ± 0.33* | -6.39 ± 0.6* |
| **Cerulean Warbler** | Parulidae | 0.36 ± 0.14* | -0.17 ± 0.12* | 1.37 ± 0.18* | 0.23 ± 0.15* | -0.4 ± 0.14* | -0.42 ± 0.14* | -3.61 ± 0.2* |
| **Hooded Warbler** | Parulidae | 0.63 ± 0.1* | -0.28 ± 0.08* | -0.05 ± 0.08 | 0.63 ± 0.1* | -0.02 ± 0.1 | -0.02 ± 0.09 | -1.09 ± 0.09* |
| **Yellow-rumped Warbler** | Parulidae | -0.11 ± 0.12 | -0.04 ± 0.11 | -0.16 ± 0.12* | -1.14 ± 0.17* | -0.08 ± 0.12 | 0.02 ± 0.11 | -3.57 ± 0.19* |
| **Yellow-throated Warbler** | Parulidae | 0.65 ± 0.18* | 0.06 ± 0.11 | -0.25 ± 0.11* | 1.39 ± 0.17* | -0.06 ± 0.16 | -0.45 ± 0.18* | -3.74 ± 0.21* |
| **Blackburnian Warbler** | Parulidae | 0.28 ± 0.1* | -0.45 ± 0.1* | -0.21 ± 0.09* | -1.37 ± 0.12* | 0.03 ± 0.1 | -0.07 ± 0.09 | -2.25 ± 0.12* |
| **Magnolia Warbler** | Parulidae | 0.13 ± 0.12* | -0.21 ± 0.12* | -0.24 ± 0.12* | -1.74 ± 0.18* | -0.4 ± 0.13* | -0.17 ± 0.13* | -3.64 ± 0.2* |
| **Palm Warbler** | Parulidae | 0.07 ± 0.35 | 0.21 ± 0.29 | -0.17 ± 0.33 | 0.53 ± 0.41* | 0.24 ± 0.35 | -0.07 ± 0.44 | -7.93 ± 1.09* |
| **Chestnut-sided Warbler** | Parulidae | 0.03 ± 0.09 | 0.1 ± 0.08* | 0.32 ± 0.09* | -1.3 ± 0.13* | 0.03 ± 0.09 | 0.61 ± 0.08* | -2.08 ± 0.11* |
| **Pine Warbler** | Parulidae | -0.33 ± 0.13* | -0.34 ± 0.11* | -0.38 ± 0.09* | 0.75 ± 0.12* | -0.32 ± 0.12* | -0.63 ± 0.14* | -2.89 ± 0.13* |
| **American Redstart** | Parulidae | 0.12 ± 0.08* | -0.15 ± 0.07* | 0.83 ± 0.08* | -0.53 ± 0.08* | -0.18 ± 0.08* | -0.48 ± 0.08* | -0.91 ± 0.08* |
| **Blackpoll Warbler** | Parulidae | -0.19 ± 0.18* | -0.01 ± 0.17 | 0.27 ± 0.18* | 0.1 ± 0.2 | 0.23 ± 0.19* | -0.41 ± 0.2* | -4.51 ± 0.29* |
| **Cape May Warbler** | Parulidae | 0.02 ± 0.31 | 0.18 ± 0.23 | -0.14 ± 0.27 | 0.24 ± 0.32 | 0.25 ± 0.28 | -0.5 ± 0.36* | -7.24 ± 0.82* |
| **Black-throated Green Warbler** | Parulidae | 0.17 ± 0.09* | -0.48 ± 0.08* | -0.5 ± 0.08* | -0.25 ± 0.09* | 0.1 ± 0.09* | 0.03 ± 0.08 | -1.16 ± 0.09* |
| **Eastern Bluebird** | Turdidae | -0.05 ± 0.12 | 0.68 ± 0.09* | 0.28 ± 0.1* | 0.46 ± 0.12* | 0.05 ± 0.11 | -0.01 ± 0.11 | -2.57 ± 0.14* |
| **Red-breasted Nuthatch** | Sittidae | -0.2 ± 0.13* | -0.36 ± 0.16* | -0.83 ± 0.13* | -0.92 ± 0.18* | -0.4 ± 0.14* | 0.95 ± 0.13* | -4.1 ± 0.24* |
| **White-breasted Nuthatch** | Sittidae | -0.03 ± 0.1 | 0.04 ± 0.08 | 0.22 ± 0.08* | 0.34 ± 0.09* | -0.03 ± 0.09 | -0.18 ± 0.09* | -1.69 ± 0.1* |
| **Brown-headed Nuthatch** | Sittidae | 0.14 ± 0.44 | 0.34 ± 0.35 | -0.34 ± 0.39 | 2.05 ± 0.73* | 0.3 ± 0.47 | -0.3 ± 0.8 | -8.97 ± 1.37* |
| **Yellow-bellied Sapsucker** | Picidae | 0.07 ± 0.13 | -0.06 ± 0.12 | 0.01 ± 0.13 | -2.26 ± 0.23* | -0.34 ± 0.14* | -0.47 ± 0.15* | -4.36 ± 0.28* |
| **Pine Siskin** | Fringillidae | -0.36 ± 0.28* | 0.26 ± 0.31 | -0.26 ± 0.27* | 0 ± 0.53 | -0.04 ± 0.35 | 1.56 ± 0.39* | -7.16 ± 0.77* |
| **American Goldfinch** | Fringillidae | 0.01 ± 0.1 | 0.4 ± 0.08* | 0.2 ± 0.09* | -0.01 ± 0.09 | -0.3 ± 0.1* | 0 ± 0.08 | -1.01 ± 0.08* |
| **Chipping Sparrow** | Passerellidae | -0.13 ± 0.09* | 0.3 ± 0.07* | 0.04 ± 0.07 | 0 ± 0.08 | -0.18 ± 0.09* | -0.45 ± 0.08* | -0.97 ± 0.08* |
| **Barred Owl** | Strigidae | -0.22 ± 0.17* | -0.15 ± 0.16 | 0.04 ± 0.16 | -0.01 ± 0.19 | -0.12 ± 0.17 | 0.03 ± 0.18 | -3.82 ± 0.19* |
| **Carolina Wren** | Troglodytidae | 0.29 ± 0.1* | 0.21 ± 0.08* | 0 ± 0.08 | 1.48 ± 0.11* | -0.16 ± 0.1* | -0.15 ± 0.1* | -1.66 ± 0.1* |
| **Winter Wren** | Troglodytidae | 0.14 ± 0.14 | -0.35 ± 0.16* | -0.65 ± 0.14* | -1.77 ± 0.22* | 0.11 ± 0.14 | 0.43 ± 0.13* | -4.6 ± 0.3* |
| **American Robin** | Turdidae | 0.1 ± 0.08* | 0.3 ± 0.07* | 0.13 ± 0.07* | -0.75 ± 0.08* | -0.1 ± 0.08* | -0.04 ± 0.07 | -0.1 ± 0.07* |
| **Eastern Kingbird** | Tyrannidae | 0.11 ± 0.19 | 0.39 ± 0.14* | -0.01 ± 0.16 | -0.33 ± 0.18* | -0.05 ± 0.18 | -1.18 ± 0.24* | -4.67 ± 0.34* |
| **Yellow-throated Vireo** | Vireonidae | -0.2 ± 0.12* | 0 ± 0.09 | 0.51 ± 0.1* | 0.48 ± 0.12* | -0.07 ± 0.11 | -0.84 ± 0.12* | -2.7 ± 0.14* |
| **Eastern/Western Warbling Vireo** | Vireonidae | 0.33 ± 0.19* | 0.42 ± 0.15* | 0.16 ± 0.19 | -1.35 ± 0.26* | 0.05 ± 0.18 | -1.82 ± 0.33* | -5.4 ± 0.45* |
| **Red-eyed Vireo** | Vireonidae | 0.18 ± 0.1* | -0.14 ± 0.08* | 0.14 ± 0.08* | 0.15 ± 0.1* | 0.07 ± 0.1 | -0.58 ± 0.09* | 1.47 ± 0.09* |
| **Philadelphia Vireo** | Vireonidae | 0.25 ± 0.33 | -0.04 ± 0.3 | -0.15 ± 0.33 | -0.49 ± 0.4* | -0.1 ± 0.33 | -0.6 ± 0.46* | -6.58 ± 0.7* |
| **Blue-headed Vireo** | Vireonidae | 0.1 ± 0.08* | -0.35 ± 0.08* | -0.21 ± 0.07* | -0.3 ± 0.08* | -0.07 ± 0.08 | 0.78 ± 0.08* | -1.03 ± 0.08* |
| **White-throated Sparrow** | Passerellidae | -0.06 ± 0.17 | 0.14 ± 0.14 | 0.03 ± 0.16 | -0.94 ± 0.21* | 0.12 ± 0.17 | -0.9 ± 0.21* | -6.02 ± 0.5* |

Trait Association with Responses to Forest Structure

Table S4. Trait associations with the vertical and horizontal structure relationships of breeding season birds in Bird Conservation Region 28. (All) indicates associations across all species in the community. (+) indicates associations for birds that respond positively to vertical or horizontal structure. (-) indicates associations for birds that respond negatively to vertical or horizontal structure. Bolding and asterisks indicate no overlap between 95% CI and zero.

| **Trait** | **Vertical (All)** | **Vertical (+)** | **Vertical (-)** | **Horizontal (All)** | **Horizontal (+)** | **Horizontal (-)** |
| --- | --- | --- | --- | --- | --- | --- |
| **Inverts.** | 0.02 ± 0.08 | **0.09 ± 0.07*** | 0 ± 0.1 | -0.03 ± 0.1 | 0.03 ± 0.06 | **0.1 ± 0.07*** |
| **Plants** | 0.03 ± 0.05 | 0.04 ± 0.06 | -0.01 ± 0.09 | -0.01 ± 0.07 | 0.03 ± 0.04 | 0 ± 0.04 |
| **Seeds** | -0.03 ± 0.06 | -0.01 ± 0.05 | 0.02 ± 0.05 | 0.04 ± 0.08 | 0 ± 0.03 | **0.05 ± 0.04*** |
| **Canopy** | **0.13 ± 0.07*** | **0.14 ± 0.03*** | **0.05 ± 0.05*** | -0.01 ± 0.09 | 0.04 ± 0.07 | **0.04 ± 0.03*** |
| **Ground** | 0.04 ± 0.08 | **0.12 ± 0.05*** | 0 ± 0.05 | 0.03 ± 0.1 | **0.09 ± 0.05*** | 0.02 ± 0.04 |
| **Understory** | **0.09 ± 0.07*** | **0.09 ± 0.03*** | **0.06 ± 0.06*** | 0.02 ± 0.09 | **0.05 ± 0.05*** | -0.02 ± 0.03 |
| **HWI** | -0.03 ± 0.06 | **-0.04 ± 0.04*** | 0 ± 0.04 | 0.04 ± 0.07 | **0.05 ± 0.02*** | -0.04 ± 0.07 |
| **Mass** | -0.02 ± 0.07 | -0.06 ± 0.09 | 0.03 ± 0.09 | 0.05 ± 0.09 | -0.01 ± 0.04 | **0.17 ± 0.06*** |
| **Nest Height** | -0.03 ± 0.06 | -0.03 ± 0.04 | -0.03 ± 0.03 | 0.01 ± 0.07 | **0.06 ± 0.05*** | -0.01 ± 0.03 |

Extreme Species’ Responses to Vertical and Horizontal Structure

Figure S6. Responses of each of the 106 bird species analyzed to vertical forest structure (median FHD) and horizontal forest structure (SD FHD). Species labeled in blue have responses in 80^th^ percentile or above to either horizontal or vertical forest structure. Species with less extreme relationships to forest structure are in grey.

Figure S7. Difference in occurrence probability (Pr(Occ)) predictions on the probability scale (0 to 1) between models with and without structure predictors at regional (a; across BCR28) and finer (b; < 50km) scales. Predictions are for Northern parula (*S. americana*). Green pixels (no difference) indicate areas in which structure predictors explain little variation in occurrence. Red pixels (large difference, positive or negative) indicate areas where structure explains substantial variation in occurrence. Though structure predictors have minimal impact on occurrence predictions at regional scale (a), they exert much greater influence at finer scales (b).

Species-Specific Importance of Structure

Figure S8. Distribution of the importance of vertical structure across the entire avian community (106 species) of BCR28. Although the median importance of the vertical structure predictor (median FHD) was only 1.6%, some species (e.g., Northern Parula) experienced much higher importance, reflecting the nuanced importance of structure in our analysis.
